# The imprint of the Slave Trade in an African American population: mitochondrial DNA, Y chromosome and HTLV-1 analysis in the Noir Marron of French Guiana

**DOI:** 10.1186/1471-2148-10-314

**Published:** 2010-10-19

**Authors:** Nicolas Brucato, Olivier Cassar, Laure Tonasso, Patricia Tortevoye, Florence Migot-Nabias, Sabine Plancoulaine, Evelyne Guitard, Georges Larrouy, Antoine Gessain, Jean-Michel Dugoujon

**Affiliations:** 1 Laboratoire d'Anthropobiologie Moléculaire et Imagerie de Synthèse, CNRS and Université Paul Sabatier, FRE2960, Toulouse, France; 2 Unité d'Epidémiologie et Physiopathologie des Virus Oncogènes, Institut Pasteur, Paris, France; 3 Institut de Recherche pour le Développement (IRD) UMR 216 Mère et enfant face aux infections tropicales & Faculté de Pharmacie, Université Paris Descartes, Paris, France; 4 Institut National de la Santé et de la Recherche Médicale, U980, Paris, France

## Abstract

**Background:**

Retracing the genetic histories of the descendant populations of the Slave Trade (16^th^-19^th ^centuries) is particularly challenging due to the diversity of African ethnic groups involved and the different hybridisation processes with Europeans and Amerindians, which have blurred their original genetic inheritances. The Noir Marron in French Guiana are the direct descendants of maroons who escaped from Dutch plantations in the current day Surinam. They represent an original ethnic group with a highly blended culture. Uniparental markers (mtDNA and NRY) coupled with HTLV-1 sequences (*env *and LTR) were studied to establish the genetic relationships linking them to African American and African populations.

**Results:**

All genetic systems presented a high conservation of the African gene pool (African ancestry: mtDNA = 99.3%; NRY = 97.6%; HTLV-1 e*nv *= 20/23; HTLV-1 LTR = 6/8). Neither founder effect nor genetic drift was detected and the genetic diversity is within a range commonly observed in Africa. Higher genetic similarities were observed with the populations inhabiting the Bight of Benin (from Ivory Coast to Benin). Other ancestries were identified but they presented an interesting sex-bias. Whilst male origins spread throughout the north of the bight (from Benin to Senegal), female origins were spread throughout the south (from the Ivory Coast to Angola).

**Conclusions:**

The Noir Marron are unique in having conserved their African genetic ancestry, despite major cultural exchanges with Amerindians and Europeans through inhabiting the same region for four centuries. Their maroon identity and the important number of slaves deported in this region have maintained the original African diversity. All these characteristics permit to identify a major origin located in the former region of the Gold Coast and the Bight of Benin; regions highly impacted by slavery, from which goes a sex-biased longitudinal gradient of ancestry.

## Background

The genetic dispersal that occurred during the Slave Trade remains complex, due to the large diversity of populations involved. Modern African American populations show this genetic diversity, inherited from African, European and Amerindian populations. Nine to ten million Africans were deported to the American colonies during the 16^th^-19^th ^centuries. In the Guianese regions [1], between Brazil and Venezuela, approximately 400,000 slaves arrived to work in the plantations from 1604 to 1815 for mainly Dutch, but also English, French and Portuguese Jew settlers. Although many of these slaves were shipped by the Dutch *West Indische Compagnie *(WIC, 1620-1674), which controlled several African trading posts notably in the Bight of Benin, most of them were sold by independent slavers who traded along the Atlantic coast of Africa. Historical reports show widespread origins of the enslaved working force in Guiana, ranging over the Atlantic African coast from Senegal to Angola: 12% of the slaves came from Senegambia (present-day Senegal, Guinea-Bissau, Guinea), Sierra Leone and the *Windward Coast *(present-day Liberia and a part of the Ivory Coast), 56% from the Gold Coast (the remaining part of the Ivory Coast and Ghana) and the Bight of Benin (present-day Togo, Benin and a part of Nigeria), 5% from the Bight of Biafra (the remaining part of present-day Nigeria, Cameroon, Equatorial Guinea and Gabon), and 28% from South West Africa (present-day Angola) [2]. The slavers took advantage of this high cultural diversity, by breaking all ethnic and familial networks and by maintaining a sex-ratio (2/3 men and 1/3 women) to prevent rebellions during the *Middle Passage *(the crossing of Atlantic Ocean) and in the plantations.

Despite these precautions, many slaves managed to escape from European oppression once on American soil, a phenomenon known as *marooning*. In Dutch Guiana, these maroons took refuge in the equatorial Amazonian forest, and reconstructed entire communities known as the *Noir Marron *(or *Bushinengué*) [3]. These escapes were favoured by conflicts between the Dutch and French, as was observed during the French embargo of Dutch Guiana in 1712. They rapidly adapted themselves to this new environment, to represent a real threat for the colonial power, forcing the government to sign peace treaties. Between 1760 and 1809, six Noir Marron communities were officially recognized: Saramaka, Ndjuka, Aluku, Paramaka, Matawai and Kwinti [3]. From the beginning of the 19^th ^century to 1986 and the Surinamese civil war, they were often evicted from their territories and many of them were forced to take refuge in French Guiana. Despite these fluctuating conditions of life, the Noir Marron communities have prospered and their culture has asserted itself. Although the maroon identity is their cornerstone, cultural exchanges with Europeans and Amerindians have been intense. Large influences are detectable, for example, in their languages composed of English, Dutch, Portuguese or Arawak words, and in the structure of their villages, which are inherited from Amerindian knowledge. Today, living in Surinam and French Guiana, 50,000 individuals constitute one of the last known American maroon society [3], and contribute to the large ethnic diversity of African American populations.

The complex variety of African American communities has been well-studied, notably with genetic data revealing a high diversity among them. These populations can be highly admixed, as an Afro-Brazilian group from the State of Saõ Paulo whose origins are at: 26% African, 62% European and 12% Amerindian [4]. They can have a balanced admixed profile, as the Brazilian community of Cameta: 53% African, 24% European and 23% Amerindian [5]. But some of them show a relatively preserved African ancestry, as the Gullah/Geechee in United States: 96% African and 3% European and 1% Amerindian [6]. Furthermore, these studies have shown that gene-flow within each group is often sex-biased, adding another level of complexity, as in the Afro-Venezuelian community of Birongo which has a maternal African inheritance of 100% and a paternal European ancestry of 93% [5]. Concerning the Noir Marron population, a preliminary anthropological study on the Gm allotypic system showed a high degree of conservation of the African genetic contribution (95.1%) and very low levels of European (2.6%) and Amerindian (1.7%) contributions. This contradicts expectations based on their cultural exchanges and emphasises the importance of the maroon identity which has shaped their profile of admixture [7]. Moreover, this seems to constitute an original genetic characteristic in comparison with other African American populations inhabiting Latin America. Although the Gm system has a high power of discrimination between continental populations, it is limited for finer-scale analyses [8]. These original findings have to be confirmed by more powerful tools. Consequently, three genetic markers have been analysed in the present study, to obtain a better picture of the genetic structure of the Noir Marron. Uniparental lineages were determined through the analysis of mitochondrial DNA (mtDNA) and non-recombinant Y chromosome (NRY) haplotypes. Through the large number of African American and African populations typed for these systems, updated by original data from the Ivory Coast and Benin, the data available are informative to identify the ancestry of each haplotype observed [9,10]. A third particular genetic system, the HTLV-1 retrovirus, has been explored because of its interest in human populations [11,12]. Due to its ability to integrate itself in human genome, its low mutation rate and its mode of transmission (mainly mother-to-child), it represents a relevant marker for infected groups. For some genotypes and especially concerning the Long Terminal Repeat (LTR) region, the phylo-geographical tree is highly discriminative [12]. This study is the first to combine these three genetic systems to study an African American population. In obtaining a better understanding of the genetic structure of the Noir Marron in French Guiana, the aim of this study is dual: (i) to confirm or not the highly preserved African gene pool obtained from the Gm allotypic system, which contradicts the cultural exchanges between the Noir Marron, Amerindians and Europeans, and (ii) to retrace the origin of this community by estimating the contribution of the historical African areas of slavery.

## Methods

### Population Samples

#### Noir Marron from French Guiana

**Figure 1:**
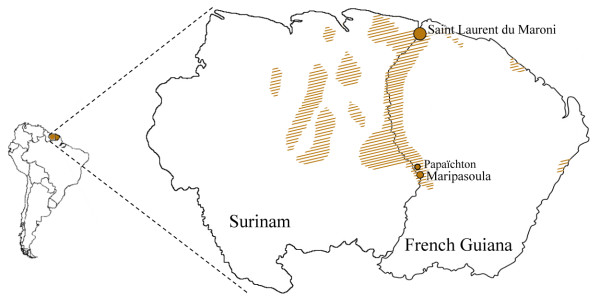
**Geographic location of the Noir Marron communities sampled**. The brown hatched areas locate the Noir Marron communities in Surinam and in French Guiana. The brown points represent the communities sampled and their relative size depends to the number of sampled individuals.

One hundred and forty-two DNA extracts by phenol-chloroform protocol from Peripheral Blood Buffy Coat (PBBC) from individuals belonging to the four Noir Marron populations in French Guiana have been collected previously, during former collaborative studies in Saint-Laurent du Maroni, Maripasoula and Papaichton in the Maroni river region [13-16]: 80 Ndjuka, 41 Saramaka, 10 Aluku and 11 Paramaka. Genealogical data has been collected upon three generations to exclude related individuals. The locations of these populations are shown in Figure 1. Informed consent was obtained from all participants and the study was performed after authorisation from the Commission Nationale de l'Informatique et des Libertés (CNIL), the Comité Consultatif de Protection des Personnes dans la Recherche Biomédicale (Necker Hospital, Paris), l'Agence Française de Sécurité Sanitaire des Produits de Santé (AFSSAPS) and the Comité de Protection des Personnes Sud-Ouest et Outremer III. Because of the sampling heterogeneity and the non-significant genetic differences observed for the Gm system among the Noir Marron communities [7], a Fst test on uniparental markers, using Arlequin v.3.11 software [17], confirmed their genetic homogeneity (Fst < 0.05, p-value = 0.05, data not shown). Thus, they are considered as a whole group in this study.

#### Benin

**Figure 2:**
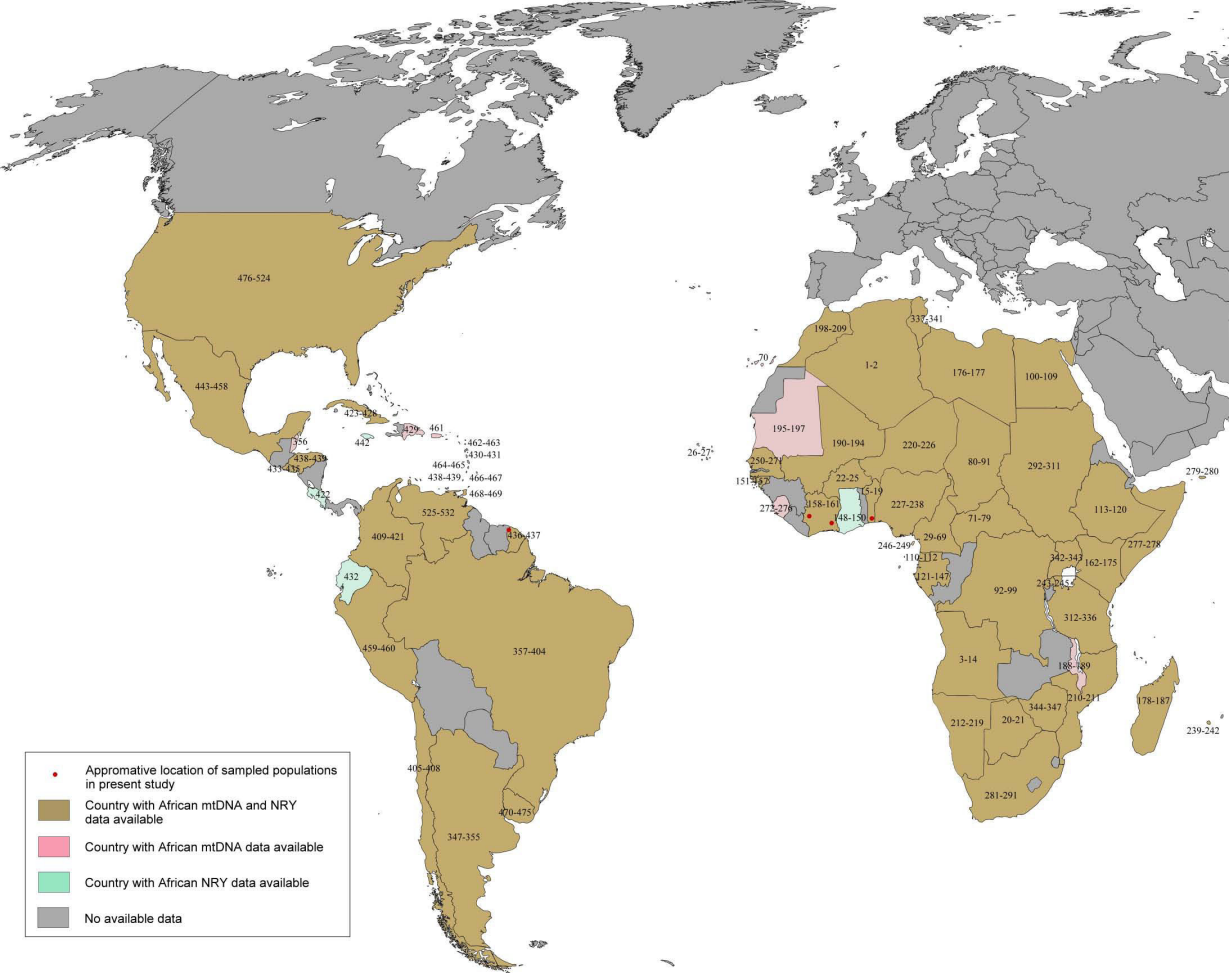
**Geographic locations of populations analysed in the study**. Each number refers to a unique group characterised by its ethnic name, country, the genetic system studied and the author of the corresponding study. The nomenclature and the references are available in Additional file 1.

The Bight of Benin, also known as the "Slave Coast", was one of the African areas most impacted by slavery between the 16^th ^and 19^th ^centuries, since more than 2,340,000 individuals were deported from its coast [2]. Thousands of people were enslaved each year and sold to European slavers in trading posts, such as in Whydah. A large part of the wealth of the Dahomey and Oyo kingdoms was based on this trade [1]. Present-day Benin is composed of more than forty different ethnic groups, the most numerous being the Fon. In an aim to enlarge the databases of this crucial region, our sampling was composed of blood samples from 82 Fon and 68 Beninese, belonging to diverse ethnic groups subjected to slavery, such as the Yoruba, Tofin and Goun. Informed consent was obtained from all participants and the study was authorised by the ethics committee of the Faculté des Sciences de la Santé at the Université d'Abomey-Calavi in Benin. The locations of these populations are shown in Figure 2.

#### Ivory Coast

This region was extensively used for slaves after the 18^th ^century. Belonging to the historical areas of the Windward Coast and the Gold Coast, approximately 700,000 Africans living in this region were deported to the Americas during the Triangular Trade [1]. Today, about 60 ethnic groups constitute the cultural diversity of the Ivory Coast. Among them, blood samples from 128 Ahizi and 62 Yacouba were collected filling a lack in the available databases. All samples were obtained with the informed consent of the participants. The locations of these two populations are shown on Figure 2.

### Laboratory methods

#### mtDNA

Maternal lineages were determined by a two-step procedure. The entire HVS-I (positions 16012-16400), the intermediate region (positions 16401-72) and part of HVS-II (positions 73-263) of the mtDNA control region were sequenced following the protocol described previously [18]. A first alignment step with rCRS [19] was made using BioEdit v.7.0.9.0, to determine a preliminary haplogroup. Twenty-seven informative Single Nucleotid Polymorphisms (SNPs) on the coding region (positions 10873, 13276, 13789, 13470, 10810, 12950, 3594, 13710, 10400, 9818, 11899, 12049, 14088, 13485, 14034, 13803, 15939, 4158, 3693, 13958, 10086, 8618, 14905, 4218, 2352, 750, 7851) were typed by minisequencing SNaPshot^® ^(PE, Applied Biosystems). All data were obtained on an ABI PRISM 3730 sequencer (PE, Applied Biosystems). The final haplogroup assignment was obtained by the most recent mtDNA phylogeny [10].

#### Y-Chromosome

NRY were determined by two types of markers. Seventeen Short tandem repeats (STRs) were typed using the *AmpFLSTR Yfiler*^® ^kit (PE, Applied Biosystems). Twenty-four Unique Event polymorphisms (UEPs; SRY 10831, M213, M9, M70, M22, Tat, 92R7, M173, P25, M96, M35, M78, M81, M123, M34, M17, M18, M73, M37, M63, M126, M153, M160, SRY 2627) were typed following the protocol published previously [20]. An additional set of nine UEPs (M33, U174, M191, M75, U209, M2, P2, M91, M60) has been designed to precise the haplogroup assignment. All data were read on an ABI PRISM 3730 sequencer and analysed with Genemapper v.4.0 (PE, Applied Biosystems). The YAP analysis has been obtained following Hammer and Horai [21]. The haplogroup assignment follows the most recently updated NRY phylogeny [9].

#### HTLV-1

The overall HTLV-I seroprevalence in the Noir Marron population in French Guiana is about 6% in women and 4% in men [13,22-25]. Determination of HTLV-1 genomic subtype in *env *and LTR regions, which are used as markers of the migration of infected populations, was performed for 23 samples of HTLV-1 infected Noir Marron individuals representative of the main Noir Marron communities: Saramaka (n = 6), Ndjuka (n = 6), Aluku (n = 5) and Paramaka (n = 6) (Genbank accession number: GU725032-GU725054) and compared with the corresponding database available in Genbank.

High molecular-weight DNA was extracted from peripheral blood buffy-coats using the QIAamp DNA Blood Mini Kit (Qiagen GmbH, Hilden, Germany). All samples were firstly determined to contain amplifiable DNA after being amplified by PCR for human β-globin. Five hundred nanograms, quantified by spectrophotometry, of each DNA sample was then subjected to two series of PCR to obtain the complete long terminal repeat (LTR) (755-bp) and a 522-bp region of the *env *gene, as previously described [26,27]. To prevent false-positive reactions, all pre- and post-PCR operations were performed in separate facilities. The complete LTR was obtained for eight individuals while the 522-bp *Env *fragment was obtained for the 23 samples tested. The amplified products of the appropriate size were cloned, sequenced and phylogenetically analysed as described [28,29].

### Data Analyses

All summary statistics for mtDNA and NRY haplotype variation, Tajima's D and Fu's Fs tests were calculated using the ARLEQUIN 3.11 software package [17]. Four databases were compiled from published studies, updated with data from the African populations of Benin and Ivory Coast analysed in the present study. The African mtDNA database was composed of 170 populations representing 8727 HVS-I and 3500 HVS-II haplotypes associated with their corresponding haplogroup assignment. The African NRY database was composed of 145 populations representing 8909 individuals typed for UEP informative for the haplogroup assignment and 1200 Y-STR profiles. As the Y- SNP haplogroup information is lacking in some African regions, it was statistically inferred from the Y-STR data available, as previously explained [30]. For some analyses, the African databases were divided into nine groups according to their historical region of slavery described by H.S. Klein [1], the genetic coherence and published genetic studies [2,31]: North Africa (Algeria, Canary Island, Egypt, Mauritania, Morocco), Windward Coast, Senegambia and Sierra Leone (Cabo Verde, Guinee-Bissau, Mali, Senegal, Sierra Leone), Gold Coast and Bight of Benin (Benin, Burkina Faso, Ivory Coast), Bight of Biafra (Cameroon, Central African Republic, Chad, Equatorial Guinea, Gabon, Nigeria, Niger, Sao Tome), South West Africa (Angola, Cabinda, Democratic Republic of Congo), South Africa (Botswana, Malawi, Namibia, Zambia, Zimbabwe), South East Africa (Mozambique), East Africa (Ethiopia, Kenya, Rwanda, Somalia, Uganda, Sudan, Tanzania), and Pygmies. The mtDNA and NRY databases of African American and urban hybridised populations were composed of 95 and 90 populations, respectively. Due to the heterogeneous resolution of haplogroup assignment among populations, forming a non-relevant database, only the admixture rates of continental populations (European, Amerindian and African), given in published studies, have been considered. Despite this bias, as African ancestry is relatively common in all African Americans [1,32], their discrimination is mostly due to differential contribution of non-African gene pools. All populations considered in the present study are located on Figure 2 and complete references are available in Additional file 1.

Haplotype networks were generated for mtDNA haplogroups L2a* and L1c*, and for the NRY haplogroup E1b1a* via the median-joining algorithm of Network v.4.5.1.6 http://www.fluxus-engineering.com from the Noir Marron data and all African and African American comparable data. To obtain the most parsimonious networks the reticulation permissivity was set to zero. Data were pre-processed using the star contraction option in Network v.4.5.1.6 [33]. For the mtDNA data, hypermutable sites were identified by post-processing using the Steiner (MP) algorithm within Network 4.5.1.6, and removed from the analysis [34]. Because of the high level of reticulation in the E1b1a* sample, Y-STR loci were subdivided into three mutation rate classes based on observed STR allelic variance and weighted as follows: 4 (low) for DYS391, DYS392; 2 (intermediate) for DYS389I, DYS389II, DYS19, DYS393, DYS390; or 1 (high) for DYS385a/b [35].

Cross-population comparisons of maternal and paternal lineages based on the frequency of haplogroups or rates of continental ancestry common to all samples in the database were performed using ARLEQUIN 3.11 [17]. The significance of Fst values is given for p-values under a threshold of 0.05. All results obtained for the comparison between the Noir Marron and each population of the database were graphically plotted on a map using Surfer v.8.0, using the location of each population given in the corresponding study. Factorial Correspondence Analysis (FCA) based on mtDNA and Y-chromosome haplogroup frequencies were performed using XLstat v.7.5.2. Analyses of molecular variance (AMOVA) were performed with ARLEQUIN 3.11 [17]. Admixture estimates were calculated by two different methods. The first, based on haplotypic homology (up to 99% of homology), was calculated by the percentage of shared lineages (LS) between the Noir Marron and each compared group [36]. Haplotype comparisons were performed from HVS-I mtDNA sequences (16030-16360) and NRY core haplotypes (DYS19, DYS389I, DYS389II, DYS390, DYS391, DYS392, DYS393, DYS385a/b) to obtain the most relevant results from the compiled databases. The second estimator, *mY*, was calculated with the ADMIX2.0 program [37]. Both mtDNA-based and NRY-based estimates were calculated from haplogroup frequencies without taking into account molecular distances between haplogroups. The parental populations were chosen among the groups that presented a Fst value, obtained by the AMOVA for the comparison with the Noir Marron, lower than an threshold fixed at 0.1.

Concerning HTLV-1 *Env *and LTR phylogenetic analysis, the phylogenetic trees were generated using the Neighbor-Joining method performed in the PAUP v.4.0b10 program using representative HTLV-1 sequences available in Genbank, including four *env *sequences and one LTR sequence typed in Noir Marron individuals of French Guiana [13]. The strains were aligned with the DAMBE v.4.2.13 program and the final alignment was submitted to the Modeltest v. 3.6 program to select, according to the Akaike Information Criterion (AIC), the best model to apply to phylogenetic analyses. Confidence levels were estimated with the distance NJBOOT program (1,000 replicates).

## Results

### mtDNA

#### Genetic diversity

A total of 78 different mtDNA haplotypes were characterised among the 142 Noir Marron individuals (GenBank accession number: GU807605 - GU808086; Additional file 2). Statistics estimating the sequence diversity were relatively high (H = 0.988 ± 0.003 ; π = 0.019 ± 0.009; θk = 72.859; Table 1) and within the range found in other African American groups and Sub-Saharan populations [32,38]. Although the Fu's test is not significant (Fs= -23.987 (0.001); Table 1), this diversity is correlated with a significant value obtained for Tajima's test, revealing a population expansion (D = -0.549 (0.357); Table 1). Considering the differences between the number of individuals in each Noir Marron community, all results are relatively similar.

**Table 1:** Summary statistics estimating the mtDNA genetic diversity of the Noir Marron.

Population	N	k	H (SD)	θk (95% CI)	θs (SD)	π (SD)	Tajima's D (P)	Fu's Fs (P)
Aluku	10	9	0.977 (± 0.054)	38.775 (9.402 - 169.084)	15.553 (± 6.606)	0.019 (± 0.010)	-0.075 (0.494)	-0.693 (0.302)
Ndjuka	80	50	0.983 (± 0.006)	60.691 (38.367 - 96.897)	17.499 (± 4.766)	0.019 (± 0.009)	-0.549 (0.357)	-17.139 (0.001)
Paramaka	11	7	0.911 (± 0.077)	9.023 (2.907 - 29.191)	14.846 (± 6.322)	0.019 (± 0.011)	0.145 (0.620)	1.899 (0.820)
Saramaka	41	23	0.975 (± 0.013)	28.055 (14.525 - 55.171)	16.512 (± 5.232)	0.020 (± 0.010)	-0.084 (0.538)	-2.178 (0.236)
Total Noir Marron	142	78	0.988 (± 0.003)	72.859 (51.408 - 103.439)	18.668 (± 0.622)	0.019 (± 0.009)	-0.549 (0.357)	-23.987 (0.001)

N: number of sequences; k: number of different haplotypes; H: Haplotype diversity; θ: mutation drift statistic calculated from the number of different haplotypes (θk) and segregating sites (θs); π: nucleotide diversity

**Figure 3:**
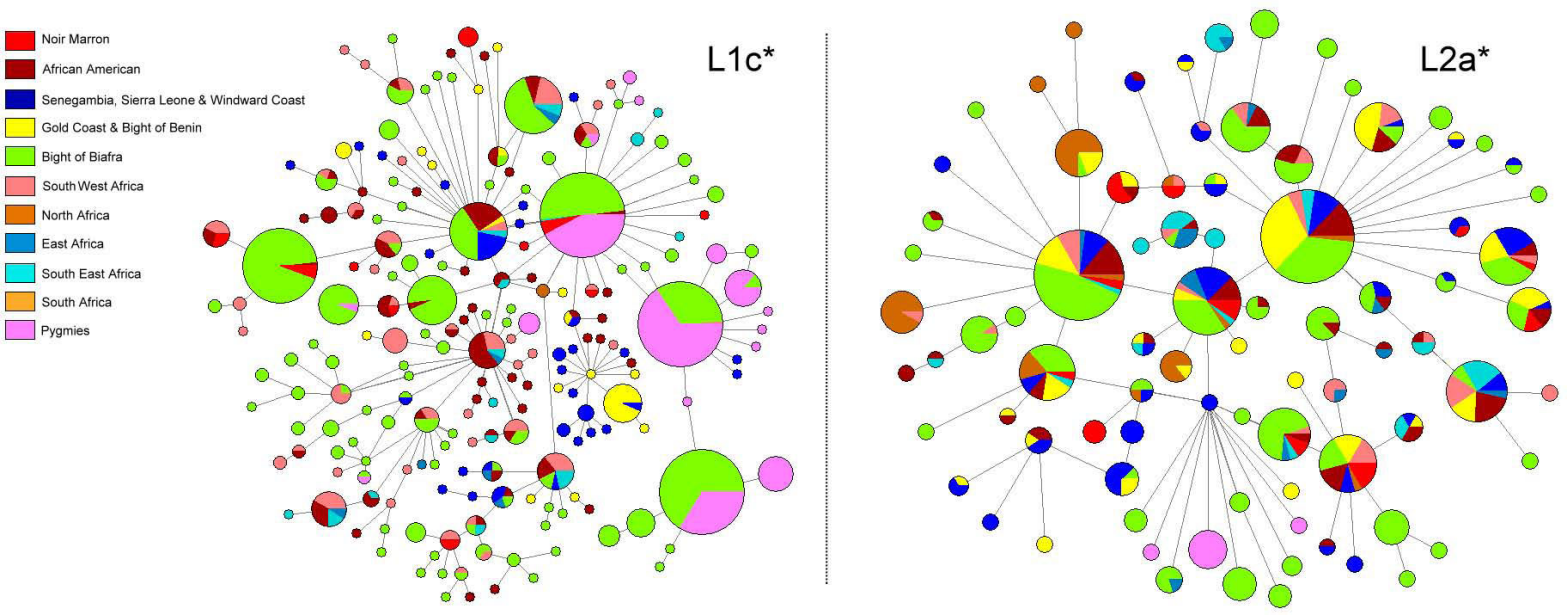
Median-joining phylogenetic trees of Noir Marron, African and African American mitochondrial HVS-I haplotypes belonging to haplogroups L1c* and L2a*.

All mtDNA haplotypes were phylogenetically identified following the latest classification [10]. 99.3% of them belonged to the major African haplogroup L* (Additional file 2). The highest percentages were observed for L2a* (22.5%) and L1c (19.0%), which are widely present in Africa [31]. Phylogenetic trees of these sub-haplogroups show no Noir Marron founder haplotype, but an integration of all haplotypes within the African and African American diversity (Figure 3). The sub-divisions L2a1 (14.1%) and L1c1 (12.7%) were found more in West African populations [31]. This West African link was also suggested by the presence of L1b (14.1%), L3e2 (7.0%), L3d (3.5%) and L3f1 (3.5%). Other L sub-haplogroups are more widely found in Africa but present low frequencies in the Noir Marron sample. A unique European haplogroup was observed through the characterization of U5b1c, which is present in the South of Europe [39]. With the aim to evaluate the maternal contribution of Africans, Europeans and Amerindians, the mtDNA admixture ratio was calculated following the geographical origin of each haplogroup. It revealed an African contribution of 99.3% and a European contribution of 0.7%, whereas no Amerindian contribution was detectable.

#### Population cross-comparisons

**Figure 4:**
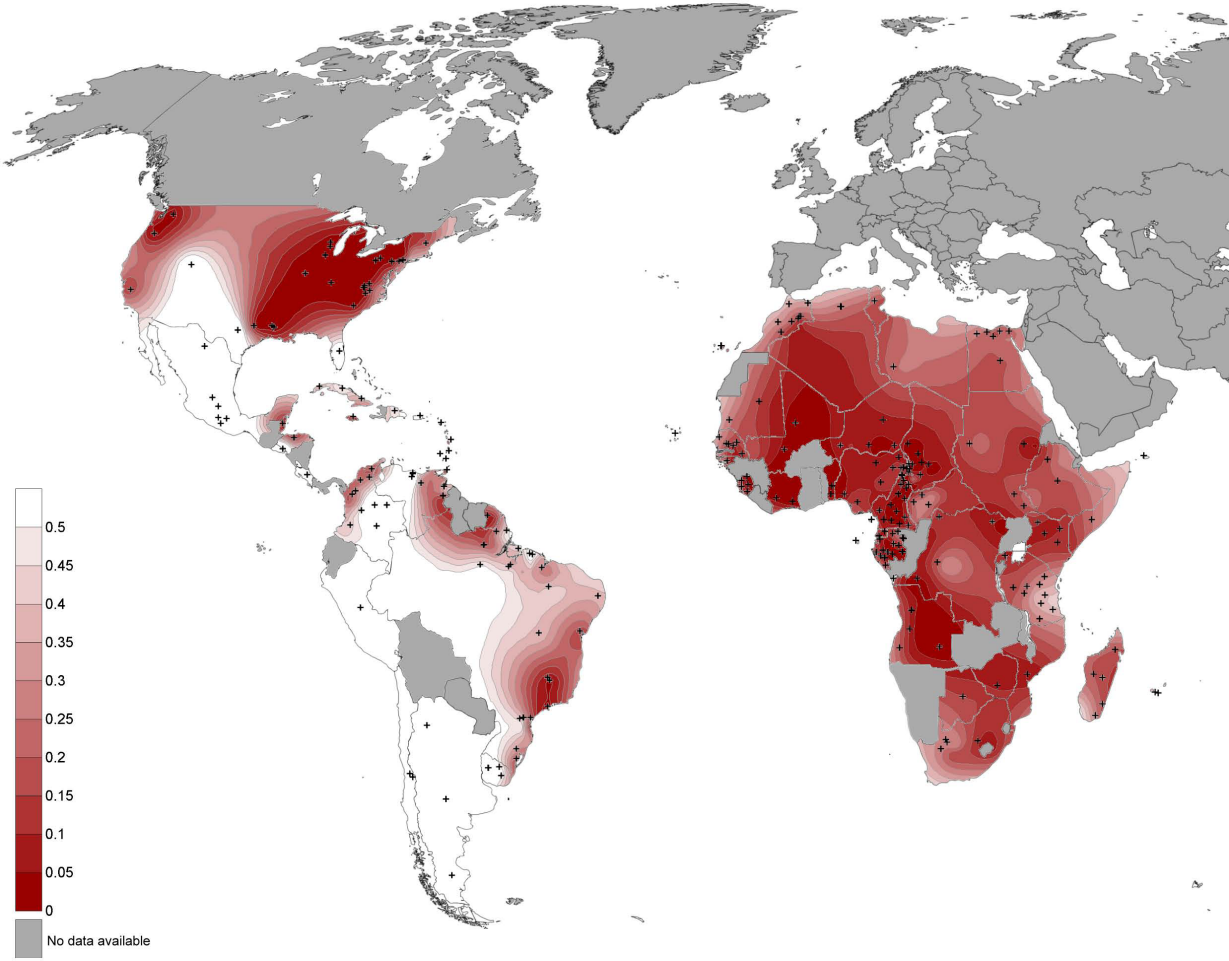
**Map of Fst values obtained for the pairwise comparison of the Noir Marron maternal lineages with those of Africans, African Americans and urban Americans**. The red colour scale represents the Fst values calculated between the mtDNA data of the Noir Marron and the populations of interest represented by black crosses.

The maternal genetic diversity of the Noir Marron was compared to other American African and African communities using a population pairwise Fst comparison. All Fst values obtained are plotted graphically on Figure 4. Among the 95 African American and American urban groups, only 24 presented low differentiations with the Noir Marron (Fst < 0.05). The majority inhabit the United States, especially the East Coast [6,40-43]. The remaining populations are the Garifunas in Belize [44], some English-speaking Caribbean groups such as St Kitts, St Vincent and St Lucia [45], Afro-Venezuelians in Curiepe [5] and the black communities of Porto Alegre, Rio de Janeiro in Brazil [46,47]. The 71 maternal lineages of other African American or urban hybridised communities were highly different from the Noir Marron (Fst > 0.25), such as the neighbouring Afro-Brazilian groups, notably due to their Amerindian ancestry [5,47-51].

Thus, the mtDNA pattern of the Noir Marron is closer to that of Africans than of most African Americans. This maternal African ancestry showed a low divergence with 41% of African groups (Fst < 0.05). Many of them are located in West Africa and South West Africa, such as Senegal [52], Cabo Verde [53], Sierra Leone [54], Guinea-Bissau [55], Mali [41], Ivory Coast (present study), Burkina Faso [56], Benin [57] (present study), Niger [56], Cameroon [58-63], Chad [59], Gabon [62], Equatorial Guinea [64], Angola [65] and Cabinda [66]. However, inside this large area, three clusters of high genetic divergence from the mtDNA gene pool were observed identifying the Pygmy communities of Cameroon [61,62], Gabon [62] and Central African Republic [63,67], the Fang from Equatorial Guinea [68] and the Mbuti from the Democratic Republic of Congo [69]. High divergences were also present in North African, East African, South East African and South African populations, even though some groups have low differences, as one Somalian population [56], one Sudanese population [70], one Namibian population [63], one Rwandan group [71] and three Bantu-speaking groups located in the Democratic Republic of Congo [69], Uganda [72] and Kenya [56].

**Figure 5:**
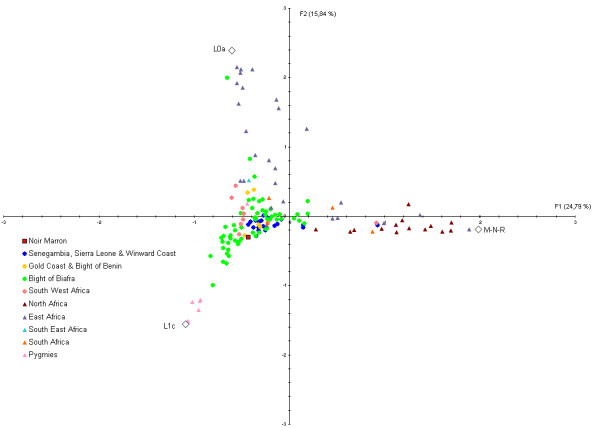
**Plot showing the 1^st ^and 2^nd ^principal component of the FCA computed from the mtDNA haplogroup frequencies of the Noir Marron and African populations**. Square point represents the Noir Marron population, circle points the West African and South West African populations, and all other groups are represented by triangular points. The colours identify the groups considered in the present study.

A Factorial Correspondence Analysis (FCA) was realised to look at the mitochondrial relationships between the Noir Marron and 170 African populations (Figure 5). 40.6% of the genetic variance is represented by Factors 1 and 2. The haplogroups M-N-R, L1c and L0a contribute significantly (contribution>5%) to Axis 1 and haplogroups L0a and L1c to Axis 2 (contribution>5%). The Noir Marron are clustered in West African and South West African populations historically enslaved, whereas North Africans, South Africans, East Africans and populations from South East Africa are peripheral to this group. This result is consistent with the results of the population pairwise Fst comparison.

An AMOVA based on the clustering of African populations by historical regions of slavery and genetic coherence showed that 10.4% of the variance lies between groups, 11.1% among populations within groups and 78.6% within populations (p values < 0.01; Additional file 3). Despite this low significance, high values were found between the Noir Marron and the East Africans, South Africans, North Africans and Pygmies (0.13≤Fst≤0.26), whereas they seem to be genetically closer to populations in the groups "Windward Coast, Senegambia and Sierra Leone", "Gold Coast and Bight of Benin", "Bight of Biafra", "South West Africa" and "South East Africa" (Fst≤0.07, Table 2).

**Table 2:** Estimators of shared maternal ancestries of the Noir Marron.

	Windward Coast, Senegambia and Sierra Leone	Gold Coast Bight of Benin	Bight of Biafra	South West Africa	North Africa	East Africa	South East Africa	South Africa	Pygmies
N pop (N indiv)	20 (1604)	7 (440)	60 (2361)	9 (625)	18 (1223)	33 (1365)	10 (425)	5 (165)	8 (519)
Fst (versus Noir Marron)	0.03	0.03	0.07	0.06	0.26	0.13	0.07	0.14	0.23
Shared Lineages rate (LS)	0.13	0.29	0.19	0.26	-	-	0.14	-	-
Admixture rate (mY)	0 (± 0.17)	0.64 (± 0.20)	0.13 (± 0.16)	0.23 (± 0.17)	-	-	0 (± 0.18)	-	-

Estimates and standard deviations (shown between parentheses) were computed from 1000 bootstraps replications. N indiv: number of individuals; N pop: number of populations; LS: Shared Lineages analysis (see [36]); *mY: *estimator of admixture (see [37]); dashes represent groups that were not included in the calculation of ancestry

#### Admixture analysis

All mtDNA data of the five African groups presenting Fst values lower than the chosen threshold (Fst < 0.1) were used to evaluate the maternal African admixture percentage of the Noir Marron community (Table 2). Both estimators, LS and *mY *rates, identify a major origin in the "Gold Coast and Bight of Benin" (LS = 29%; mY = 64%). Two other important ancestries are detected in "South West Africa" (LS = 26%; mY = 23%) and in the "Bight of Biafra" (LS = 19%; mY = 13%). The two last regions present lower probabilities of ancestry: "Windward Coast, Senegambia and Sierra Leone" (LS = 13%; mY = 0%) and "South East Africa" (LS = 14%; mY = 0%).

### Y-Chromosome

#### Genetic diversity

**Figure 6:**
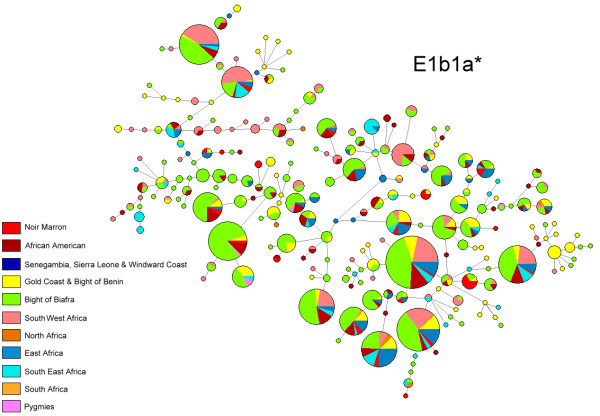
Median-joining phylogenetic trees of Noir Marron, African and African American Y-chromosome STR haplotypes belonging to haplogroups E1b1a*.

Among the 42 Noir Marron typed for NRY, 36 different haplotypes were detected (YHRD accession Number YA003610-YA003615; Additional file 4). The genetic diversity shows a high-level (H = 0.990 ± 0.008), similar to that found in other African American groups and Sub-Saharan populations [73]. All haplotypes were grouped using the latest NRY phylogeny [9]. 90.5% belong to the major African haplogroup E1b1* with notably 88% of the sub-division E1b1a*, which is more frequent in West Africa [74]. The phylogenetic tree of this sub-haplogroup shows no Noir Marron founder haplotype, but an integration of all haplotypes within the African and African American diversity (Figure 6). Three occurrences of African haplogroups A and B, more frequent in South West Africa and South Africa, especially in Pygmy and Khoisan populations, were detected [9,75]. One individual was positively typed for the R1b sub-haplogroup which is as frequent in Europe as in Cameroon [76,77]. The probable continental paternal ancestry was estimated following the geographical origin of each haplogroup. It shows an African contribution of 97.6%, a European contribution of 2.4% and no Amerindian origin.

#### Population cross-comparisons

**Figure 7:**
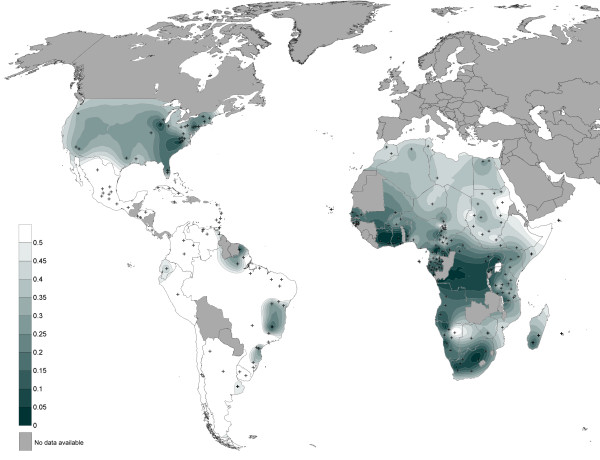
**Map of Fst values obtained for the pairwise comparison of the Noir Marron paternal lineages with those of Africans, African Americans and urban Americans**. The green colour scale represents the Fst values calculated between the NRY data of the Noir Marron and the populations of interest, which are represented by black crosses.

As for the maternal lineage, the paternal gene pool of the Noir Marron was compared to those of other African Americans and Africans (Figure 7). Only three of the 90 African Americans showed low Fst values (Fst < 0.05): a community from Illinois [78] and Baltimore in United States [43], and one from Ribeirão Preto in Brazil [5]. All others presented significant differentiation from the Noir Marron. Moreover, Fst values exceeded 0.5 in 47 of them, due to their European inheritance, for example the African Americans of North East Brazil [5,48,79-81]. The preserved African ancestry of the Noir Marron paternal lineage leads them to be genetically closer to African groups.

The NRY gene pool of the Noir Marron showed low Fst values with only 10% of the African groups; the majority located in the Ivory Coast (present study), Ghana [82], Benin (present study) and in Cameroon [82,83], and with one from Gabon [77], Angola [66], Namibia [82], Zimbabwe [82] and South Africa [82]. The other groups: North Africans, East Africans, South East Africans, Pygmies [76,77,82] and other South Africans, notably the Khoisan groups [76,82], presented high divergence (Fst>0.25). The paternal ancestry of the Noir Marron is observed to be less widespread than the maternal ancestry.

**Figure 8:**
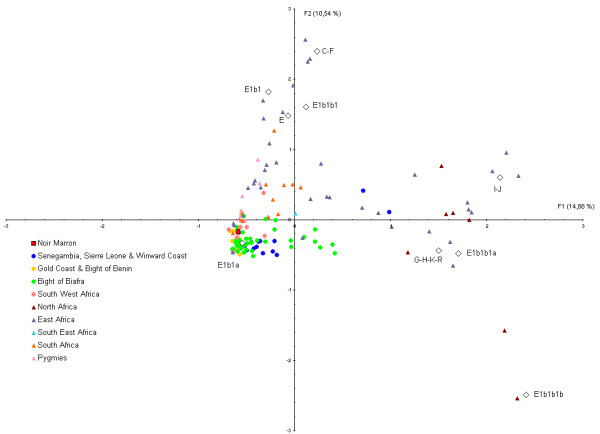
**Plot showing the 1^st ^and 2^nd ^principal component of the FCA computed from the NRY haplogroup frequencies of the Noir Marron and African populations**. Square point represents the Noir Marron population, circle points the West African and South West African populations, and all other groups are represented by triangular points. The colours identify the groups considered in the present study.

A FCA was computed to plot the Noir Marron and African genetic NRY diversity (Figure 8). A total of 25.4% of the variance is represented on Axis 1 and 2. The haplogroups G-H-K-R, E1b1b1b, E1b1b1a, E1b1a and IJ contribute significantly to Axis 1 (contribution>5%) and haplogroups E*, E1b1a, E1b1, CF, E1b1b1b and E1b1b1 to Axis 2 (contribution>5%). As observed for mtDNA, the Noir Marron are clustered in a group composed predominantly of West African and South West African populations, while other African populations are peripheral to this group.

The clustering of African populations by historical regions of slavery showed no genetic significance, due to the low genetic divergence between West African groups, as suggested by previous studies on NRY data [76,77]. Ten percent of the variance lies between groups, 25.7% among populations within groups, and 64.3% within populations (p values < 0.01; Additional file 3). However, confirming the results of the population pairwise Fst comparison, the lowest values were obtained between the Noir Marron and "Gold Coast and Bight of Benin", "Windward Coast, Senegambia and Sierra Leone", "Bight of Biafra", "South West Africa" and "South Africa" (Fst≤0.08; Table 3).

**Table 3:** Estimators of shared paternal ancestries of the Noir Marron.

	Windward Coast, Senegambia and Sierra Leone	Gold Coast Bight of Benin	Bight of Biafra	South West Africa	North Africa	East Africa	South East Africa	South Africa	Pygmies
N pop (N indiv)	13 (182)	10 (197)	42 (1321)	8 (236)	7 (234)	39 (462)	5 (291)	13 (54)	9 (127)
Fst (versus Noir Marron)	0.08	0.07	0.07	0.04	0.25	0.14	0.16	0.07	0.31
Shared Lineages rate (LS)	0.25	0.28	0.19	0.13	-	-	-	0.16	-
Admixture rate (mY)	0.26 (± 0.28)	0.74 (± 0.19)	0 (± 0.35)	0 (± 0.20)	-	-	-	0 (± 0.15)	-

Estimates and standard deviations (shown between parentheses) were computed from 1000 bootstraps replications. N indiv: number of individuals; N pop: number of populations; LS: Shared Lineages analysis (see [36]); *mY: *estimator of admixture (see [37]); dashes represent groups that were no included in the calculation of ancestry

#### Admixture analysis

All NRY data of the five African groups presenting Fst values lower than the chosen threshold (Fst < 0.1) were used to evaluate the paternal African admixture percentage of the Noir Marron community (Table 3). As for mtDNA analysies, both estimators, LS and *mY *rates, identify a major origin in the "Gold Coast and Bight of Benin" (LS = 28%; mY = 74%). One other non negligible ancestry is located in "Windward Coast, Senegambia and Sierra Leone" (LS = 25%; mY = 26%). The remaining regions show lower probabilities of ancestry: "Bight of Biafra" (LS = 19%; mY = 0%) "South West Africa" (LS = 13%; mY = 0%).

### HTLV-1

#### Sequence analysis

The analysis of the *env *522-bp fragments of the 23 new HTLV-1 Noir Marron strains indicated neither deletion nor insertion compared with the ATK-1 reference strain. Comparison of the 23 strains indicated that four sequences and two pairs were identical. All together, the 23 strains showed a nucleotidic interstrain difference ranging from 0-1.5% and an amino acid divergence ranging from 0-1.7%. Nevertheless, 17 strains were closely related with 0-0.8% of nucleotide differences on 522 nucleotides and 0-1.7% at the amino acid level.

In parallel, LTR sequences obtained from eight representative individuals of the four Noir Marron communities: Saramaka (2), Ndjuka (2), Aluku (2) and Paramaka (2) were analysed and exhibited 0-2.4% divergence at the nucleotide level. Interestingly, six strains were closely related with only 0-0.7% nucleotidic divergence for the 757-bp LTR fragment.

#### Phylogenetic analysis

**Figure 9:**
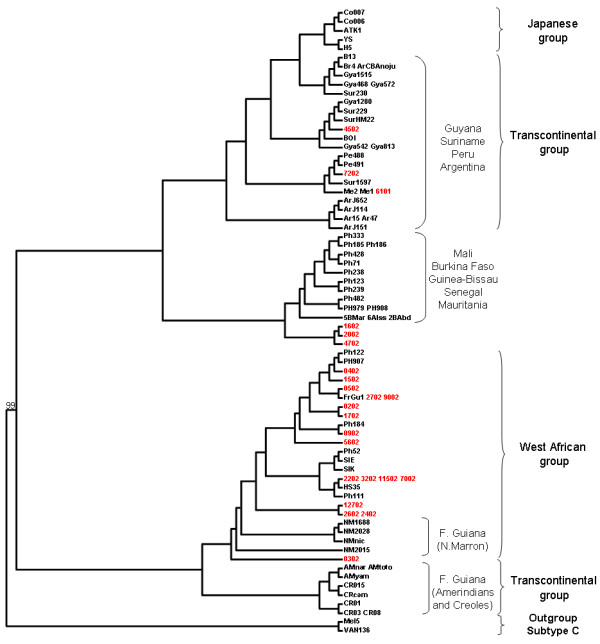
***Env *phylogenetic tree generated using the Neighbor-Joining method performed in the PAUP program (v4.0b10) on a 519-bp fragment of the *Env *gene using 75 HTLV-1 available sequences available in Genbank**. The new Noir Marron data are coded in red. The Noir Marron data already published are coded "NM". The Mel5 and VAN136 strains were used as out-group. The HTLV-1 strains were aligned with the DAMBE program (version 4.2.13). The final alignment was submitted to the Modeltest program (version 3.6) to select, according to the Akaike Information Criterion (AIC), the best model to apply to phylogenetic analyses. The selected model was the GTR. Bootstrap support (1,000 replicates) is noted on the branch of the tree.

Phylogenetic analyses were performed comparing these 23 new sequences with 70 representatives e*nv *gene sequences available in GenBank of the HTLV-1 Cosmopolitan A subtype, as well as sequences obtained from individuals originating from Melanesian C subtype, used as an outgroup (Figure 9). Firstly, all sequences belonged to the large cosmopolitan subtype and none were related to other subtypes, especially other African ones (B or D), which are mainly found in Central Africa (data not shown). Secondly, among the 23 Noir Marron HTLV-1 *env *strains, 17 clustered in the same large "West Africa" subgroup, clustering strains from Senegal, Burkina Faso and Ivory Coast. Three strains (1602, 2002 and 4702) constitute a cluster related to strains originating from Mali, Burkina-Faso, Guinea-Bissau, Senegal and Mauritania, while the remaining three (4502, 7202 and 6101) were related to South American strains from Peru, Surinam and Guyana. Worth noting is that most of the LTR sequences (6/8) analysed belonged to the "West Africa" subgroup, while the remaining two clustered in the "Transcontinental" subgroup (Additional file 5).

## Discussion

### An original African American population

The African American community is composed of highly diverse ethnic groups in terms of their historical, cultural and biological inheritances. Each of them has their specific identity. Being one of the last known American maroon community, the Noir Marron are an important part this diversity [3]. A previous analysis of the Gm system showed their preserved African gene pool (>95%), revealing a first insight into their genetic originality in comparison with other neighbouring African American groups [7]. The present study, exploring three different genetic systems, strengthens this conclusion. Although each parental lineage presents European and/or Amerindian contribution, the bulk (>95%) of their genetic diversity is inherited from African ancestors. Indeed, 99.3% of the mtDNA genetic profile of the Noir Marron comes from Africa, whereas the paternal African ancestry increases to 97.6% and the HTLV-1 strains, probably of African origin, represent 6/8 for the LTR and 20/23 for the *env *region. Thus, despite four centuries in America, neighboured by European settlers and Amerindian ethnic groups with whom the Noir Marron made cultural exchanges [3], none or restricted gene flow has influenced their African genetic identity. This emphasises the role of the maroon identity in shaping their genetic profile. Although exchanges were necessary for the survival of the community, the ethnic integrity of the maroon society is characterised by a struggle against foreign threats, and above all the struggles of former slavers. Thus, interethnic couples, which effectively exist as between the Aluku and the Wayana [3], would contradict this cultural rule. This does not mean that the Noir Marron have been genetically isolated in the equatorial forest as revealed by their genetic diversity (H_mtDNA _= 0.988; H_NRY _= 0.990). Comparable to the values of African populations, these values reflect the mass arrival of slaves into the Guianas over four centuries, an important proportion of which increased the number of the Noir Marron. Genetic diversities do not seem to have been impacted by demographic crises or geographical isolations which could have occurred during the history of Noir Marron which was punctuated by many conflicts (D = -0.55 (0.357); Table 1). Although some communities were briefly reduced to a hundred individuals, as the Aluku were [3], the constant flow of new maroons balanced the genetic lost (Figures 3 and 6). Forming sustainable groups, still increasing in number, their adaptation to the Guianese context has been facilitated. Their maroon identity, their adaptation to the Amazonian environment and the relatively important number of immigrants explain why the Noir Marron, who today constitute 30,000 individuals in French Guiana, have conserved their African genetic inheritance.

These characteristics are all the more interesting when compared with other African American groups. The composition of the uniparental haplogroups of this African gene pool is close to observations in other African American populations, notably because of the high frequencies of L2a (22,5%) and L1c (19%) for the maternal lineage and E1b1a (88%) for the paternal lineage (Figures 3 and 6), such in African American in the United States (L1c = 11%; L2a = 19%; E1b1a = 62%) or in Afro-Brazilian in Porto Alegre (L1c = 14%; L2a = 17%; E1b1a = 44%) [40,46,84]. As most of the slavers traded along the Atlantic coast of Africa, the average composition of each lineage is similar among the African American group. If this ancestral genetic pool was effectively common, the evolution of each one has produced highly divergent patterns. The population pairwise Fst comparisons of both uniparental systems showed that the majority of African American groups presented significant genetic differences from the Noir Marron (Fst>0.25; Figures 4 and 7). The discrepancies are mainly due to European and/or Amerindian contributions, which are most often sex-biased [85]. This preferential gene flow is partly due to the importance of European male migration and an easier acceptance of Amerindian women in tri-hybridised communities [86]. The African inheritance of the Noir Marron, consequently not sex-biased, is peculiar in the African American landscape. Even their neighbouring African American communities are much more hybridised, as observed in North East Brazil [5,47,48,50,79-81]. If this characteristic is mainly shaped by their maroon identity, it is surprising to notice that other maroon groups present an admixed and sex-biased genetic profile, such as the Maroons in Jamaica (African maternal lineage: 87%; African paternal lineage: 58%) [45]. Even the Curiau *quilombo*, which could have originated from the same plantations as the Noir Marron, present a highly hybridised genetic profile (African maternal lineage: 54%; African paternal lineage: 37%) [81]. Furthermore, most maroon groups have lost a part of their genetic diversity after genetic drift due to their relative isolation, as has occurred in the Garifuña in Honduras (H_mtDNA _= 0.897) [87] and in the Angolares in the African island of São Tomé (H_mtDNA _= 0.919) [88,89]. Thus, the Noir Marron are also genetically original within the maroon populations cluster. The reason for this difference can be enlightened by examining the groups presenting similar patterns in both lineages (Fst < 0.15). They are all located on the East coast of the United States, such as the Gullah/Geechee in South Carolina [43,78]. Although these groups never present an estimated African ancestry as high as in the Noir Marron, they present a low sex-bias. A historical cultural phenomenon can be evoked to link this common pattern. Although assimilation of Amerindian and African women was encouraged as a strategy for the occupation in Portuguese colonies [90], tolerated in Spanish territories [1], it was not the case in French, English and Dutch colonies [91,92]. Such cultural traits have favoured inter-ethnic admixture in the first case and separated communities in the second. The common behaviours in these colonies have caused a low level of sex-biased admixture, bringing the gene pools of the Noir Marron and the African Americans of the East coast of the United States closer. The combination of the maroon identity in the former Dutch colony has preserved the African genetic diversity of the Noir Marron ancestors.

### Retracing the African origin

The exceptional conservation of the African genetic diversity allows a relevant picture of the genetic inheritance of slaves that were deported to the Guianas to be determined. If the African inheritance is genetically marked by the predominance of L* haplogroup in maternal lineages, E1b1* in paternal lineages and HTLV-1 A strains of African origin, the detailed examination of each of these components gives a more precise location of their ancestries [31,76]. Indeed, a close link with West African populations, also suggested by the Gm system analysis [7], is identifiable through the relatively high frequencies of some mtDNA sub-haplogroups as L2a1 (22.5%), L1b (14.1%), L3d (3.5%), L3f1 (3.5%), NRY sub-haplogroups as E1b1a7* (33.3%) and E1b1a8* (21.4%) [31,76]. Most of the HTLV-1 Noir Marron strains analysed were clustered with West African strains originating from the Ivory Coast, Ghana, Senegal and Burkina-Faso (Figure 9). Secondly, genetic relationships between the Noir Marron and South West African populations can be distinguished. Although no HTLV-1 B and D subtypes (Central African genotypes) were detected among the studied samples, the observed frequencies of mtDNA sub-haplogroups L1c1 (12.7%) and L3e2 (7.4%), and the NRY sub-haplogroup B (4.7%) [9,31,62] sign the link between the Noir Marron and South West Africa. These two genetic affinities were confirmed by cross-comparisons between the gene pools of the Noir Marron and each African population (Figures 4 and 7). The ethnic groups inhabiting West and South West Africa presented the lowest genetic divergence (Fst < 0.15) compared with other African groups, at the noticeable exception of the Pygmy communities, the Fang and some groups from Senegal, Chad and Niger. North Africans, East Africans and Khoisans (Fst>0.25) have not contributed to the Noir Marron gene pool, confirming that the presence of NRY haplogroups A and B are probably of South West African inheritance. The West and South West African mixed-ancestry of the Noir Marron is attested by FCA performed for each lineage, plotting the Noir Marron diversity inside a cluster of these populations (Figures 5 and 8). As hypothesised, the populations located in the historical regions of slavery are the most probable Noir Marron ancestors.

Despite the fact that the relative genetic homogeneity within these populations make any grouping non-significant, as reported in many studies [41,93], historical data give a strong significance to the clustering because of their importance during the Atlantic Slave Trade. All estimators of ancestry, for both uniparental systems, state the Gold Coast and the Bight of Benin as the major origin of the Noir Marron's ancestors (mtDNA: LS = 0.29; *mY *= 0.64; NRY: LS = 0.28; *mY *= 0.74; Tables 2 and 3). A region also suggested by the phylogenetic analyses of the HTLV-1 strains (Figure 9 and Additional file 5). These converging results are concordant with historical records showing that this region was the port of departure of slaves who were deported to the Guianas [2]. Almost 224,000 individuals, natives of the Gold Coast and the Bight of Benin, were sold as slaves to work in the Guianese plantations. Their relative importance has largely imprinted the genetic background of the Noir Marron but also their culture. For example, Akan words are still present in their vocabulary; their pantheon is largely inherited from those present in populations in Ghana; funeral rites are common with Fanti-Ashanti customs [3]. Thus, the Noir Marron culture and genes have kept the traces of individuals coming from the Gold Coast and the Bight of Benin, a region largely impacted by the Atlantic Slave Trade.

As highlighted by historical data, slaves never came from a unique region and the Noir Marron gene pool has also kept this characteristic. Indeed, other ancestries are detected in uniparental systems, in a sex-biased manner, contradictory to the Gold Coast and the Bight of Benin origin. The majority of the remaining maternal ancestry is located in the Bight of Biafra (LS = 0.19; *mY *= 0.13; Tables 2) and in South West Africa (LS = 0.28; *mY *= 0.23; Tables 2), while the largest part of the remaining paternal ancestry is located in the region of the Windward Coast, Senegambia and Sierra Leone (LS = 0.25; *mY *= 0.26; Tables 3). Thus, a sex-biased ancestry is detected in the Noir Marron gene pool. From a major origin in the Gold Coast and the Bight of Benin, a paternal gradient goes north, while a maternal gradient goes south. This divergent geographical gradient of the uniparental ancestries may be explained by regional-specific characteristics of trading during the Atlantic Slave Trade. In the Windward Coast, Senegambia and Sierra Leone, slave trade was also implied in the Trans-Saharan Slave Trade [94] in which women were more sold than men, reducing the number of women sent to the Americas. Men from these regions were judged by European settlers to be more robust than Angolans to work in the plantations, raising the demand of male slaves in these trading posts, such as in the Gorée Island. The maternal gradient, more surprisingly, may be the consequence of these practices. The rising price of male slaves from the Windward Coast, Senegambia and Sierra Leone would have forced the slavers to balance the cost of buying slaves in the south, where slave markets were created for the Atlantic Slave Trade, without competition from the Arab traders. Thus, in the Bight of Biafra and South West Africa, slavers could have bought more women to maintain a sex-ratio close to 2/3 men and 1/3 women [1].

## Conclusions

Belonging to the wide African American cultural area, the Noir Marron in French Guiana is unique due to their African gene pool. Despite four centuries neighboured by Europeans and Amerindians with whom intense cultural exchanges were made, their maroon identity has limited gene flow. The conservation of the African diversity in each genetic system studied revealed a probable non-altered inheritance from their slave ancestors. A major origin was located on the Gold Coast and in the Bight of Benin; regions highly impacted by slavery. From this region, uniparental genetic markers showed a sex-biased origin, with the remaining male ancestry located from Senegal to Benin, and the remaining female ancestry from the Ivory Coast to Angola. Different historical and cultural traits of the Slave Trade have created a differential migration of the female and male enslaved ancestors of the Noir Marron. Thus, this sex-biased African ancestry is still genetically imprinted in the Noir Marron gene pool, a characteristic that deserves to be examined in other African American groups, such as the Creoles, in order to gain a relevant picture of the dynamics of the African gene flow that occurred during the Slave Trade.

## Authors' contributions

NB carried out genetic laboratory analysis of uniparental markers, analysed the data and drafted the manuscript. OC carried out HTLV-1 genetic analysis and the phylogenetic analysis. LT contributed with the experimental design for uniparental markers. EG extracted DNA from African samples. PT and SP participated in obtaining of the Noir Marron sampling. FMN provided the Beninese sampling. JMD, AG and GL conceived and directed the study. All authors reviewed the manuscript during its drafting and approved the final version.

## Supplementary Material

Additional file 1 **References of the populations compiled in the databases used for the comparisons to Noir Marron data**. Click here for file

Additional file 2 **MtDNA haplotypes and their respective haplogroup classification found in Noir Marron, Beninese, Yacouba and Ahizi sampling**. Click here for file

Additional file 3 **AMOVA analyses performed with mtDNA and NRY data to compare the Noir Marron gene pool with the ones of the databases**. Click here for file

Additional file 4 **NRY haplotypes and their respective haplogroup classification found in Noir Marron, Beninese, Yacouba and Ahizi sampling**. Click here for file

Additional file 5 **LTR phylogenetic tree constructed by the neighbour-joining method of HTLV-1 strains in 8 Noir Marron (in red) and HTLV-1 sequences of the database**. The Noir Marron data already published are coded "NM". The H24 strain was used as out-group. The HTLV-1 strains were aligned with the DAMBE program (version 4.2.13). The final alignment was submitted to the Modeltest program (version 3.6) to select, according to the Akaike Information Criterion (AIC), the best model to apply to phylogenetic analyses. The selected model was the GTR. Bootstrap support (1,000 replicates) is noted on the branch of the tree. Click here for file
