# Supplementary material for: The imprint of the Slave Trade in an African American population: mitochondrial DNA, Y chromosome and HTLV-1 analysis in the Noir Marron of French Guiana
Source: BMC Evol Biol. 2010 Oct 19;10:314. doi: 10.1186/1471-2148-10-314 (PMC2973943; doi:10.1186/1471-2148-10-314)
Supplement: Additional file 1 — References of the populations compiled in the databases used for the comparisons to Noir Marron data. [file 1471-2148-10-314-S1.DOC]

Additional file 4a. References of the African populations compiled in the databases.

| Country | Population | Genetic System | N | Reference | Code |
| --- | --- | --- | --- | --- | --- |
| Algeria | Misc. | MT | 85 | [1] | 1 |
| Algeria | Misc. | Y | 100 | [2] | 2 |
| Angola | Cabinda | MT | 93 | [3] | 3 |
| Angola | Cabinda | Y | 8 | [3] | 4 |
| Angola | Ganguela | MT | 17 | [4] | 5 |
| Angola | Ganguela | Y | 11 | [4] | 6 |
| Angola | Kuvale | MT | 27 | [4] | 7 |
| Angola | Kuvale | Y | 24 | [4] | 8 |
| Angola | Misc. | MT | 82 | [5] | 9 |
| Angola | Misc. | Y | 27 | [4] | 10 |
| Angola | Nyaneka-Nhkumbi | MT | 86 | [4] | 11 |
| Angola | Nyaneka-Nkhumbi | Y | 61 | [4] | 12 |
| Angola | Ovimbudu | MT | 73 | [4] | 13 |
| Angola | Ovimbudu | Y | 78 | [4] | 14 |
| Benin | Fon | MT | 85 | [6] | 15 |
| Benin | Fon | MT | 82 | present study | 16 |
| Benin | Fon | Y | 3 | [7] | 17 |
| Benin | Misc. | MT | 68 | present study | 18 |
| Benin | Misc. | Y | 78 | present study | 19 |
| Botswana | Misc. | MT | 54 | [8] | 20 |
| Botswana | Misc. | Y | 3 | [9] | 21 |
| Burkina Faso | Fulbe | MT | 2 | [10] | 22 |
| Burkina Faso | Fulbe | Y | 2 | [11] | 23 |
| Burkina Faso | Mossi | Y | 6 | [11] | 24 |
| Burkina Faso | Rimaibe | Y | 6 | [11] | 25 |
| Cabo Verde | North | Y | 14 | [12] | 26 |
| Cabo Verde | South | MT | 135 | [13] | 27 |
| Cameroon | Baka Pygmy | MT | 141 | [14] | 28 |
| Cameroon | Baka Pygmy | Y | 5 | [15] | 29 |
| Cameroon | Bakaka | MT | 36 | [16] | 30 |
| Cameroon | Bakaka | Y | 17 | [17] | 31 |
| Cameroon | Bakola Pygmy | MT | 137 | [14] | 32 |
| Cameroon | Bakola Pygmy | Y | 22 | [15] | 33 |
| Cameroon | Bamileke | MT | 1 | [18] | 34 |
| Cameroon | Bamileke | Y | 6 | [7] | 35 |
| Cameroon | Bamilleke | MT | 36 | [19] | 36 |
| Cameroon | Bantu | MT | 14 | [6] | 37 |
| Cameroon | Bantu | Y | 4 | [7] | 38 |
| Cameroon | Bassa | MT | 39 | [16] | 39 |
| Cameroon | Bassa | Y | 22 | [17] | 40 |
| Cameroon | Daba | MT | 16 | [16] | 41 |
| Cameroon | Daba | Y | 4 | [11] | 42 |
| Cameroon | Ewondo | MT | 25 | [14] | 43 |
| Cameroon | Ewondo | MT | 39 | [19] | 44 |
| Cameroon | Ewondo | Y | 4 | [11] | 45 |
| Cameroon | Fali | MT | 26 | [16] | 46 |
| Cameroon | Fali | Y | 20 | [11] | 47 |
| Cameroon | Fang | MT | 39 | [14] | 48 |
| Cameroon | Fang | Y | 4 | [15] | 49 |
| Cameroon | Fulbe | Y | 27 | [11] | 50 |
| Cameroon | Mandara | MT | 31 | [16] | 51 |
| Cameroon | Mandara | Y | 28 | [17] | 52 |
| Cameroon | Misc. | MT | 12 | [10] | 53 |
| Cameroon | Misc.Adamawa | Y | 6 | [11] | 54 |
| Cameroon | Misc.Chadic | Y | 6 | [11] | 55 |
| Cameroon | Ngoumba | MT | 47 | [20] | 56 |
| Cameroon | Ngoumba | MT | 88 | [14] | 57 |
| Cameroon | Ngoumba | Y | 4 | [17] | 58 |
| Cameroon | Ngoumba | Y | 24 | [15] | 59 |
| Cameroon | Nilo-Saharian | Y | 6 | [11] | 60 |
| Cameroon | Podokwo | MT | 33 | [16] | 61 |
| Cameroon | Podokwo | Y | 6 | [17] | 62 |
| Cameroon | Tali | MT | 15 | [16] | 63 |
| Cameroon | Tali | Y | 5 | [11] | 64 |
| Cameroon | Tigar | MT | 35 | [14] | 65 |
| Cameroon | Tupuri | MT | 23 | [16] | 66 |
| Cameroon | Tupuri | Y | 15 | [17] | 67 |
| Cameroon | Uldeme | MT | 25 | [16] | 68 |
| Cameroon | Uldeme | Y | 13 | [17] | 69 |
| Canary Islands | Guanche | MT | 39 | [21] | 70 |
| Central African Republic | Baka Pygmy | Y | 3 | [17] | 71 |
| Central African Republic | Bakola Pygmy | Y | 7 | [17] | 72 |
| Central African Republic | Bantu | Y | 88 | [22] | 73 |
| Central African Republic | Biaka Pygmy | MT | 58 | [14] | 74 |
| Central African Republic | Biaka Pygmy | Y | 11 | [17] | 75 |
| Central African Republic | Lissongo | Y | 5 | [11] | 76 |
| Central African Republic | Misc. | MT | 3 | [18] | 77 |
| Central African Republic | Pygmy | MT | 10 | [23] | 78 |
| Central African Republic | Sanga | MT | 30 | [20] | 79 |
| Chad | Arabs Chad | MT | 27 | [24] | 80 |
| Chad | Arabs Shuwa | MT | 38 | [24] | 81 |
| Chad | Buduma | MT | 30 | [24] | 82 |
| Chad | Fali | MT | 40 | [24] | 83 |
| Chad | Fulani Borgor | MT | 49 | [24] | 84 |
| Chad | Fulani Tcheboua | MT | 40 | [24] | 85 |
| Chad | Hide | MT | 23 | [24] | 86 |
| Chad | Kanembu | MT | 50 | [24] | 87 |
| Chad | Kanuri | MT | 31 | [24] | 88 |
| Chad | Kotoko | MT | 56 | [24] | 89 |
| Chad | Mafa | MT | 32 | [24] | 90 |
| Chad | Masa | MT | 32 | [24] | 91 |
| Democratic Republic of Congo | Alur | Y | 3 | [17] | 92 |
| Democratic Republic of Congo | Babinga | MT | 44 | [20] | 93 |
| Democratic Republic of Congo | Bateke | MT | 50 | [20] | 94 |
| Democratic Republic of Congo | Hema | Y | 5 | [17] | 95 |
| Democratic Republic of Congo | Mbuti Pygmy | MT | 59 | [8] | 96 |
| Democratic Republic of Congo | Mbuti Pygmy | Y | 13 | [17] | 97 |
| Democratic Republic of Congo | Misc. | MT | 13 | [8] | 98 |
| Democratic Republic of Congo | Nande | Y | 2 | [17] | 99 |
| Egypt | Arab | MT | 83 | [25] | 100 |
| Egypt | Arab | MT | 101 | [26] | 101 |
| Egypt | Arab | Y | 20 | [7] | 102 |
| Egypt | Berber Siwa | MT | 78 | [27] | 103 |
| Egypt | Copte | MT | 9 | [25] | 104 |
| Egypt | Copte | MT | 101 | [27] | 105 |
| Egypt | Misc. | MT | 32 | [28] | 106 |
| Egypt | Misc. | Y | 19 | [17] | 107 |
| Egypt | Misc.Upper Egypt | MT | 24 | [28] | 108 |
| Egypt | Nubia | MT | 11 | [25] | 109 |
| Equatorial Guinea | Bubi | MT | 54 | [29] | 110 |
| Equatorial Guinea | Fang | MT | 11 | [30] | 111 |
| Equatorial Guinea | Misc. | Y | 94 | [31] | 112 |
| Ethiopia | Amhara | Y | 8 | [32] | 113 |
| Ethiopia | Amharic | MT | 251 | [33] | 114 |
| Ethiopia | Borana | MT | 1 | [10] | 115 |
| Ethiopia | Jew | Y | 7 | [11] | 116 |
| Ethiopia | Misc. | MT | 19 | [33] | 117 |
| Ethiopia | Oromo | Y | 9 | [32] | 118 |
| Ethiopia | Somali | MT | 9 | [10] | 119 |
| Ethiopia | South Semitic | Y | 8 | [17] | 120 |
| Gabon | Akele | MT | 98 | [14] | 121 |
| Gabon | Ateke | MT | 54 | [14] | 122 |
| Gabon | Babongo | MT | 45 | [14] | 123 |
| Gabon | Baka Pygmy | Y | 33 | [15] | 124 |
| Gabon | Baka-GB | MT | 39 | [14] | 125 |
| Gabon | Bakoya | MT | 31 | [14] | 126 |
| Gabon | Bekwil | Y | 5 | [15] | 127 |
| Gabon | Benga | MT | 98 | [14] | 128 |
| Gabon | Duma | MT | 93 | [14] | 129 |
| Gabon | Eshira | MT | 82 | [14] | 130 |
| Gabon | Eviya | MT | 62 | [14] | 131 |
| Gabon | Fang | MT | 66 | [14] | 132 |
| Gabon | Fang | Y | 60 | [15] | 133 |
| Gabon | Galoa | MT | 98 | [14] | 134 |
| Gabon | Kota | MT | 109 | [14] | 135 |
| Gabon | Makina | MT | 88 | [14] | 136 |
| Gabon | Mitsogo | MT | 64 | [14] | 137 |
| Gabon | Ndumu | MT | 75 | [14] | 138 |
| Gabon | Nzebi | MT | 120 | [14] | 139 |
| Gabon | Obama | MT | 47 | [14] | 140 |
| Gabon | Obamba | Y | 47 | [15] | 141 |
| Gabon | Okande | Y | 6 | [15] | 142 |
| Gabon | Orungu | MT | 41 | [14] | 143 |
| Gabon | Punu | MT | 110 | [14] | 144 |
| Gabon | Shake | MT | 94 | [14] | 145 |
| Gabon | Teke | Y | 48 | [15] | 146 |
| Gabon | Tsogo | Y | 60 | [15] | 147 |
| Ghana | Ewe | Y | 3 | [17] | 148 |
| Ghana | Fante | Y | 6 | [17] | 149 |
| Ghana | Ga | Y | 3 | [17] | 150 |
| Guinea-Bissau | Balanta | Y | 6 | [34] | 151 |
| Guinea-Bissau | Bijagos | Y | 4 | [34] | 152 |
| Guinea-Bissau | Felupe-Ejamat | Y | 6 | [34] | 153 |
| Guinea-Bissau | Fulbe | Y | 8 | [34] | 154 |
| Guinea-Bissau | Mandenka | Y | 4 | [34] | 155 |
| Guinea-Bissau | Nalu | Y | 5 | [34] | 156 |
| Guinea-Bissau | Papel | MT | 197 | [34] | 157 |
| Ivory Coast | Ahizi | MT | 128 | present study | 158 |
| Ivory Coast | Ahizi | Y | 66 | present study | 159 |
| Ivory Coast | Yacouba | MT | 62 | present study | 160 |
| Ivory Coast | Yacouba | Y | 49 | present study | 161 |
| Kenya | Bantu | MT | 42 | [6] | 162 |
| Kenya | Bantu | Y | 7 | [7] | 163 |
| Kenya | Kikuyu | MT | 4 | [10] | 164 |
| Kenya | Kikuyu | MT | 21 | [10] | 165 |
| Kenya | Kikuyu Kamba | Y | 7 | [17] | 166 |
| Kenya | Luo | Y | 5 | [17] | 167 |
| Kenya | Masai | Y | 6 | [17] | 168 |
| Kenya | Misc. | MT | 3 | [18] | 169 |
| Kenya | Sakuye | MT | 1 | [10] | 170 |
| Kenya | Somali | MT | 4 | [10] | 171 |
| Kenya | Somali | MT | 11 | [10] | 172 |
| Kenya | Swahili | MT | 200 | [35] | 173 |
| Kenya | Turkana | MT | 8 | [10] | 174 |
| Kenya | Turkana | MT | 29 | [10] | 175 |
| Lybia | Arab | Y | 63 | [36] | 176 |
| Lybia | Tuareg | MT | 129 | [37] | 177 |
| Madagascar | Antaisaka | Y | 8 | [38] | 178 |
| Madagascar | Antaisakaa | MT | 4 | [38] | 179 |
| Madagascar | Antandroy | Y | 46 | [38] | 180 |
| Madagascar | Antandroya | MT | 12 | [38] | 181 |
| Madagascar | Antanosy | Y | 47 | [38] | 182 |
| Madagascar | Antanosya | MT | 13 | [38] | 183 |
| Madagascar | Merina | MT | 5 | [38] | 184 |
| Madagascar | Merina | Y | 9 | [38] | 185 |
| Madagascar | Misc. | MT | 14 | [39] | 186 |
| Madagascar | Sihanaka Merina Betsileo Bezanozanob | MT | 10 | [38] | 187 |
| Malawi | Malawi | MT | 2 | [23] | 188 |
| Malawi | Misc. | MT | 5 | [23] | 189 |
| Mali | Bambara | MT | 19 | [40] | 190 |
| Mali | Dogon | Y | 6 | [17] | 191 |
| Mali | Malinke | MT | 61 | [40] | 192 |
| Mali | Songhai | MT | 1 | [10] | 193 |
| Mali | Tuareg | MT | 1 | [10] | 194 |
| Mauritania | Arab | MT | 1 | [26] | 195 |
| Mauritania | Maure | MT | 46 | [41] | 196 |
| Mauritania | Misc. | MT | 59 | [42] | 197 |
| Morocco | Arab | MT | 2 | [43] | 198 |
| Morocco | Arab | Y | 6 | [11] | 199 |
| Morocco | Arab Figuig | MT | 92 | [27] | 200 |
| Morocco | Berber | MT | 52 | [43] | 201 |
| Morocco | Berber | MT | 130 | [42] | 202 |
| Morocco | Berber | Y | 7 | [11] | 203 |
| Morocco | Berber Bouhria | MT | 70 | Coudray unpublished | 204 |
| Morocco | Berber Figuig | MT | 124 | [27] | 205 |
| Morocco | Berber Asni | MT | 53 | Coudray unpublished | 206 |
| Morocco | Harattine | MT | 21 | [27] | 207 |
| Morocco | Misc. | MT | 38 | [42] | 208 |
| Morocco | Saharian | MT | 19 | [42] | 209 |
| Mozambique | Misc. | MT | 187 | [44] | 210 |
| Mozambique | Misc. | Y | 144 | [45] | 211 |
| Namibia | !Kung Sekele | Y | 7 | [17] | 212 |
| Namibia | Ambo | Y | 5 | [17] | 213 |
| Namibia | Dama | Y | 10 | [17] | 214 |
| Namibia | Herero | Y | 8 | [17] | 215 |
| Namibia | Misc. | MT | 2 | [18] | 216 |
| Namibia | Nama | Y | 6 | [17] | 217 |
| Namibia | Ovambo | Y | 51 | [46] | 218 |
| Namibia | Tsumkwe San | Y | 5 | [17] | 219 |
| Niger | Fulbe | MT | 10 | [10] | 220 |
| Niger | Hausa | MT | 2 | [10] | 221 |
| Niger | Kanuri | MT | 1 | [10] | 222 |
| Niger | Songhai | MT | 9 | [10] | 223 |
| Niger | Songhai | Y | 14 | [47] | 224 |
| Niger | Tuareg | MT | 10 | [10] | 225 |
| Niger | Tuareg | MT | 15 | [10] | 226 |
| Nigeria | Effik | MT | 2 | [18] | 227 |
| Nigeria | Fulbe | MT | 6 | [10] | 228 |
| Nigeria | Fulbe | MT | 41 | [10] | 229 |
| Nigeria | Hausa | MT | 2 | [10] | 230 |
| Nigeria | Hausa | MT | 15 | [10] | 231 |
| Nigeria | Kanuri | MT | 1 | [10] | 232 |
| Nigeria | Kanuri | MT | 12 | [10] | 233 |
| Nigeria | Misc. | MT | 7 | [48] | 234 |
| Nigeria | Yoruba | MT | 21 | [10] | 235 |
| Nigeria | Yoruba | MT | 2 | [10] | 236 |
| Nigeria | Yoruba | MT | 11 | [8] | 237 |
| Nigeria | Yoruba | Y | 30 | [49] | 238 |
| Réunion Island | Malbar | MT | 144 | [50] | 239 |
| Réunion Island | Misc. | MT | 94 | [50] | 240 |
| Réunion Island | Misc. | Y | 18 | [51] | 241 |
| Réunion Island | Zarab | MT | 42 | [50] | 242 |
| Rwanda | Hutu | MT | 94 | [6] | 243 |
| Rwanda | Hutu | Y | 8 | [7] | 244 |
| Rwanda | Tutsi | Y | 6 | [7] | 245 |
| Sao Tome | Agolares | Y | 5 | [52] | 246 |
| Sao Tome | Forros | Y | 6 | [52] | 247 |
| Sao Tome | Misc. | MT | 142 | [53] | 248 |
| Sao Tome | Tongas | Y | 10 | [52] | 249 |
| Senegal | Bainouk | MT | 1 | [42] | 250 |
| Senegal | Balante | MT | 1 | [42] | 251 |
| Senegal | Bambara | MT | 5 | [42] | 252 |
| Senegal | Diola | MT | 7 | [42] | 253 |
| Senegal | Lebou | MT | 2 | [42] | 254 |
| Senegal | Malinke | MT | 1 | [42] | 255 |
| Senegal | Mandenka | MT | 119 | [54] | 256 |
| Senegal | Mandenka | Y | 4 | [17] | 257 |
| Senegal | Manyake | MT | 5 | [42] | 258 |
| Senegal | Maure | MT | 1 | [42] | 259 |
| Senegal | Mbaouin | MT | 1 | [42] | 260 |
| Senegal | Misc. | MT | 51 | [42] | 261 |
| Senegal | Misc. | Y | 7 | [32] | 262 |
| Senegal | Peul | MT | 5 | [42] | 263 |
| Senegal | Sarakhole | MT | 3 | [42] | 264 |
| Senegal | Serer | MT | 17 | [42] | 265 |
| Senegal | Serrer | MT | 21 | [42] | 266 |
| Senegal | Soce | MT | 1 | [42] | 267 |
| Senegal | Soninke | MT | 5 | [42] | 268 |
| Senegal | Tukulor | MT | 10 | [42] | 269 |
| Senegal | Wolof | MT | 83 | [42] | 270 |
| Senegal | Wolof | Y | 7 | [17] | 271 |
| Sierra Leone | Limba | MT | 49 | [55] | 272 |
| Sierra Leone | Loko | MT | 28 | [55] | 273 |
| Sierra Leone | Mende | MT | 49 | [55] | 274 |
| Sierra Leone | Misc. | MT | 101 | [56] | 275 |
| Sierra Leone | Temne | MT | 81 | [55] | 276 |
| Somalia | Misc.Danish | Y | 96 | [57] | 277 |
| Somalia | Somali | MT | 3 | [10] | 278 |
| Soqotra | Misc. | MT | 23 | [58] | 279 |
| Soqotra | Misc. | MT | 65 | [58] | 280 |
| South Africa | !Kung | Y | 8 | [11] | 281 |
| South Africa | !XunKhwe | MT | 17 | [59] | 282 |
| South Africa | Bantu | MT | 2 | [60] | 283 |
| South Africa | Khoisan | MT | 2 | [60] | 284 |
| South Africa | Khwe | MT | 6 | [61] | 285 |
| South Africa | Khwe | Y | 5 | [11] | 286 |
| South Africa | Kung | MT | 20 | [61] | 287 |
| South Africa | Misc. | MT | 4 | [62] | 288 |
| South Africa | Sotho-Tswana | Y | 8 | [17] | 289 |
| South Africa | Xhosa | Y | 7 | [17] | 290 |
| South Africa | Zulu | Y | 7 | [17] | 291 |
| Sudan | Arakien | Y | 4 | [63] | 292 |
| Sudan | Beja | Y | 6 | [63] | 293 |
| Sudan | Borgu | Y | 4 | [63] | 294 |
| Sudan | Copte | Y | 7 | [63] | 295 |
| Sudan | Dinka | MT | 46 | [25] | 296 |
| Sudan | Dinka | Y | 3 | [63] | 297 |
| Sudan | Fulani | Y | 3 | [63] | 298 |
| Sudan | Fur | Y | 4 | [63] | 299 |
| Sudan | Gaalien | Y | 9 | [63] | 300 |
| Sudan | Hausa | Y | 6 | [63] | 301 |
| Sudan | Masalit | Y | 4 | [63] | 302 |
| Sudan | Meseria | Y | 5 | [63] | 303 |
| Sudan | Nuba | Y | 4 | [63] | 304 |
| Sudan | Nubia | MT | 83 | [25] | 305 |
| Sudan | Nubia | MT | 2 | [25] | 306 |
| Sudan | Nubian | Y | 8 | [63] | 307 |
| Sudan | Nuer | MT | 11 | [25] | 308 |
| Sudan | Nuer | Y | 3 | [63] | 309 |
| Sudan | Shilluk | MT | 8 | [25] | 310 |
| Sudan | Shilluk | Y | 4 | [63] | 311 |
| Tanzania | Biaka | Y | 8 | [59] | 312 |
| Tanzania | Burunge | MT | 38 | [59] | 313 |
| Tanzania | Burunge | Y | 23 | [59] | 314 |
| Tanzania | Datog | MT | 39 | [59] | 315 |
| Tanzania | Datog | Y | 31 | [59] | 316 |
| Tanzania | Datoga | MT | 18 | [64] | 317 |
| Tanzania | Datoga | Y | 3 | [64] | 318 |
| Tanzania | Hadza | MT | 79 | [59] | 319 |
| Tanzania | Hadza | Y | 54 | [59] | 320 |
| Tanzania | Hadzabe | MT | 49 | [64] | 321 |
| Tanzania | Hadzabe | Y | 4 | [64] | 322 |
| Tanzania | Iraqw | MT | 12 | [64] | 323 |
| Tanzania | Iraqw | Y | 4 | [64] | 324 |
| Tanzania | Iraqw | Y | 5 | [17] | 325 |
| Tanzania | Mbugwe | Y | 14 | [59] | 326 |
| Tanzania | San | Y | 8 | [59] | 327 |
| Tanzania | Sandawe | MT | 82 | [59] | 328 |
| Tanzania | Sandawe | Y | 67 | [59] | 329 |
| Tanzania | Sukuma | MT | 32 | [59] | 330 |
| Tanzania | Sukuma | Y | 35 | [59] | 331 |
| Tanzania | Turu | MT | 29 | [59] | 332 |
| Tanzania | Turu | Y | 20 | [59] | 333 |
| Tanzania | WaFiome | Y | 2 | [59] | 334 |
| Tanzania | Wairak | Y | 9 | [7] | 335 |
| Tanzania | Yoruba | Y | 12 | [59] | 336 |
| Tunisia | Misc. | MT | 30 | [65] | 337 |
| Tunisia | Berber Sejenane | Y | 36 | [66] | 338 |
| Tunisia | Berber Takrouna | Y | 12 | [66] | 339 |
| Tunisia | Misc. | Y | 4 | [17] | 340 |
| Tunisia | Sfax Misc. | Y | 81 | [67] | 341 |
| Uganda | Ganda | Y | 6 | [17] | 342 |
| Uganda | Misc. | MT | 2 | [68] | 343 |
| Zimbabwe | Misc. | MT | 2 | [68] | 344 |
| Zimbabwe | Shona | MT | 38 | [69] | 345 |
| Zimbabwe | Shona | Y | 4 | [17] | 346 |

Additional file 4b. References of the African American populations compiled in the database

| Country | | Population | Genetic System | % | Reference | Code |
| --- | --- | --- | --- | --- | --- | --- |
| Argentina | | Center | MT | - | [70] | 347 |
| Argentina | | Cordoba | Y | - | [71] | 348 |
| Argentina | | Humahuaca | MT | - | [72] | 349 |
| Argentina | | Humahuaca | Y | - | [72] | 350 |
| Argentina | | La Plata | Y | - | [73] | 351 |
| Argentina | | North | MT | - | [70] | 352 |
| Argentina | | San Salvador | MT | - | [72] | 353 |
| Argentina | | San Salvador | Y | - | [72] | 354 |
| Argentina | | South | MT | - | [70] | 355 |
| Belize | | Garifuna | MT | - | [74] | 356 |
| Brazil | | Rio das Ras | Y | - | [75] | 357 |
| Brazil | | Alagoas | MT | - | [76] | 358 |
| Brazil | | Bara | Y | - | [77] | 359 |
| Brazil | | Belém General urban | MT | - | [77] | 360 |
| Brazil | | Belém General urban | Y | - | [78] | 361 |
| Brazil | | Cajueiro | MT | - | [79] | 362 |
| Brazil | | Cameta | MT | - | [80] | 363 |
| Brazil | | Cameta | MT | - | [79] | 364 |
| Brazil | | Cameta | Y | - | [80] | 365 |
| Brazil | | Curiau | MT | - | [81] | 366 |
| Brazil | | Curiau | Y | - | [81] | 367 |
| Brazil | | Kalunga | Y | - | [75] | 368 |
| Brazil | | Marajo | MT | - | [82] | 369 |
| Brazil | | Mazagão | MT | - | [82] | 370 |
| Brazil | Middle and upper-middle class, mainly whites | | MT | - | [83] | 371 |
| Brazil | Middle and upper-middle class, mainly whites | | Y | - | [84] | 372 |
| Brazil | | Misc. | MT | - | [85] | 373 |
| Brazil | | Misc. | Y | - | [77] | 374 |
| Brazil | | Misc. | Y | - | [83] | 375 |
| Brazil | | Mocambo | Y | - | [75] | 376 |
| Brazil | | Paredao | MT | - | [80] | 377 |
| Brazil | | Paredao | Y | - | [80] | 378 |
| Brazil | | Pitimandeua | MT | - | [82] | 379 |
| Brazil | | PortoAlegre | MT | - | [86] | 380 |
| Brazil | | PortoAlegre | Y | - | [80] | 381 |
| Brazil | | PortoAlegre | Y | - | [86] | 382 |
| Brazil | | Riacho de Sacutiaba | Y | - | [75] | 383 |
| Brazil | | Ribaero Preto | Y | - | [77] | 384 |
| Brazil | | RibeiraoPreto | MT | - | [80] | 385 |
| Brazil | | RibeiraoPreto | MT | - | [79] | 386 |
| Brazil | | RibeiraoPreto | Y | - | [80] | 387 |
| Brazil | | RioDeJaneiro | MT | - | [86] | 388 |
| Brazil | | RioDeJaneiro | Y | - | [86] | 389 |
| Brazil | | RioDeJaneiro | Y | - | [79] | 390 |
| Brazil | | Salvador | MT | - | [80] | 391 |
| Brazil | | Salvador | Y | - | [77] | 392 |
| Brazil | | Santarem | MT | - | [87] | 393 |
| Brazil | | Sao Gonzalo | Y | - | [77] | 394 |
| Brazil | | Sao Paulo | MT | - | Dornelas unpublished | 395 |
| Brazil | | Sao Paulo | Y | - | [88] | 396 |
| Brazil | | Sao Paulo Black | MT | - | [89] | 397 |
| Brazil | | Sao Paulo Black | Y | - | [89] | 398 |
| Brazil | | SaoPaulo | MT | - | [89] | 399 |
| Brazil | | SaoPaulo | Y | - | [89] | 400 |
| Brazil | | Tamauari | MT | - | [82] | 401 |
| Brazil | | Trombetas | MT | - | [80] | 402 |
| Brazil | | Trombetas | MT | - | [82] | 403 |
| Brazil | | Trombetas | Y | - | [80] | 404 |
| Chile | | Santiago High Status | MT | - | [90] | 405 |
| Chile | | Santiago High Status | Y | - | [91] | 406 |
| Chile | | Santiago Low Status | MT | - | [90] | 407 |
| Chile | | Santiago Low Status | Y | - | [91] | 408 |
| Colombia | | Afro | MT | - | [92] | 409 |
| Colombia | | Afro | Y | - | [93] | 410 |
| Colombia | | Afro-Colombian | Y | - | [94] | 411 |
| Colombia | | Chocó | MT | - | [92] | 412 |
| Colombia | | Medellín General urban | MT | - | [95] | 413 |
| Colombia | | Mestizo | MT | - | [96] | 414 |
| Colombia | | Misc. | MT | - | [93] | 415 |
| Colombia | | Mulattos | MT | - | [92] | 416 |
| Colombia | | Nuquí | MT | - | [96] | 417 |
| Colombia | | Providencia | MT | - | [96] | 418 |
| Colombia | | Quibdó | MT | - | [96] | 419 |
| Colombia | | San Basilio | MT | - | [96] | 420 |
| Colombia | | Misc. | Y | - | [97] | 421 |
| Costa Rica | | Misc. | Y | - | [95] | 422 |
| Cuba center | | Misc. | MT | - | [98] | 423 |
| Cuba center | | Misc. | Y | - | [98] | 424 |
| Cuba East | | Misc. | MT | - | [98] | 425 |
| Cuba East | | Misc. | Y | - | [98] | 426 |
| Cuba West | | Misc. | MT | - | [98] | 427 |
| Cuba West | | Misc. | Y | - | [98] | 428 |
| Dominican Republic | | General urban | MT | - | [99] | 429 |
| Dominica | | Misc. | MT | - | [100] | 430 |
| Dominica | | Misc. | Y | - | [100] | 431 |
| Ecuador | | African American | Y | - | [101] | 432 |
| El Salvador | | Metropolitan | Y | - | [102] | 433 |
| El Salvador | | Rural | MT | - | [103] | 434 |
| El Salvador | | Rural | Y | - | [102] | 435 |
| French Guiana | | Noir Marron | MT | - | present study | 436 |
| French Guiana | | Noir Marron | Y | - | present study | 437 |
| Grenada | | Misc. | MT | - | [100] | 438 |
| Grenada | | Misc. | Y | - | [100] | 439 |
| Honduras | | Garifuna | MT | - | [104] | 440 |
| Honduras | | Misc. | Y | - | [105] | 441 |
| Jamaica | | Misc. | Y | - | [100] | 442 |
| Mexico | | Huichol | Y | - | [106] | 443 |
| Mexico | | Mestizos | Y | - | [106] | 444 |
| Mexico | | Mestizos GD | MT | - | [107] | 445 |
| Mexico | | Mestizos SD | MT | - | [107] | 446 |
| Mexico | | Mixe | Y | - | [106] | 447 |
| Mexico | | Mixtecs | MT | - | [107] | 448 |
| Mexico | | Mixtecs | Y | - | [106] | 449 |
| Mexico | | Nahua | Y | - | [106] | 450 |
| Mexico | | Nahuas | MT | - | [107] | 451 |
| Mexico | | North-Central General urban | MT | - | [108] | 452 |
| Mexico | | Pure´pecha | Y | - | [106] | 453 |
| Mexico | | Tarahumara | Y | - | [106] | 454 |
| Mexico | | Tlapanecs | MT | - | [107] | 455 |
| Mexico | | Tzotzil | Y | - | [106] | 456 |
| Mexico | | Western Mestizos | Y | - | [106] | 457 |
| Mexico | | Zapotecs | Y | - | [106] | 458 |
| Peru | | Misc. | MT | - | [109] | 459 |
| Peru | | Misc. | Y | - | [109] | 460 |
| Puerto Rico | | General urban | MT | - | [110] | 461 |
| St. Kitts | | Misc. | MT | - | [100] | 462 |
| St. Kitts | | Misc. | Y | - | [100] | 463 |
| St. Lucia | | Misc. | MT | - | [100] | 464 |
| St. Lucia | | Misc. | Y | - | [100] | 465 |
| St. Vincent | | Misc. | MT | - | [100] | 466 |
| St. Vincent | | Misc. | Y | - | [100] | 467 |
| Trinidad | | Misc. | MT | - | [100] | 468 |
| Trinidad | | Misc. | Y | - | [100] | 469 |
| Uruguay | | Cerro Largo | MT | - | [111] | 470 |
| Uruguay | | Melo | MT | - | [112] | 471 |
| Uruguay | | Melo | Y | - | [112] | 472 |
| Uruguay | | Tacaurembó | MT | - | [107] | 473 |
| Uruguay | | Tacaurembó Low status | MT | - | [107] | 474 |
| Uruguay | | Tacaurembó Low status | Y | - | [107] | 475 |
| USA | | Arizona Mesa | Y | - | [113] | 476 |
| USA | | Arizona Phoenix | Y | - | [113] | 477 |
| USA | | Baltimore | MT | - | [9] | 478 |
| USA | | Baltimore | Y | - | [9] | 479 |
| USA | | California | MT | - | [114] | 480 |
| USA | | Chicago | MT | - | [9] | 481 |
| USA | | Chicago | Y | - | [9] | 482 |
| USA | | Connecticut | Y | - | [113] | 483 |
| USA | | Florida | MT | - | [115] | 484 |
| USA | | Florida | Y | - | [113] | 485 |
| USA | | Florida | Y | - | [114] | 486 |
| USA | | Gullah | MT | - | [116] | 487 |
| USA | | Gullah | Y | - | [116] | 488 |
| USA | | Gullah/Geechee | MT | - | [40] | 489 |
| USA | | Illinois | MT | - | [114] | 490 |
| USA | | Illinois | Y | - | [114] | 491 |
| USA | | Indiana | Y | - | [114] | 492 |
| USA | | Louisiana | MT | - | [114] | 493 |
| USA | | Louisiana | Y | - | [114] | 494 |
| USA | | Maryland | MT | - | [114] | 495 |
| USA | | Maryland | Y | - | [114] | 496 |
| USA | | MidWest | MT | - | [115] | 497 |
| USA | | Misc. | MT | - | [117] | 498 |
| USA | | Missouri | MT | - | [114] | 499 |
| USA | | Missouri | Y | - | [114] | 500 |
| USA | | New York | MT | - | [115] | 501 |
| USA | | New York | Y | - | [113] | 502 |
| USA | | North Carolina | MT | - | [9] | 503 |
| USA | | North Carolina | Y | - | [113] | 504 |
| USA | | North Carolina | Y | - | [9] | 505 |
| USA | | NYC | Y | - | [114] | 506 |
| USA | | Ohio | Y | - | [113] | 507 |
| USA | | Oregon | MT | - | [114] | 508 |
| USA | | Oregon | Y | - | [114] | 509 |
| USA | | Pennsylvania | MT | - | [114] | 510 |
| USA | | Pennsylvania | Y | - | [114] | 511 |
| USA | | Pittsburgh | MT | - | [9] | 512 |
| USA | | Pittsburgh | Y | - | [9] | 513 |
| USA | | South Carolina Columbia | MT | - | [116] | 514 |
| USA | | South Carolina Columbia | Y | - | [116] | 515 |
| USA | | South Carolina Low Country | MT | - | [116] | 516 |
| USA | | South Dakota | Y | - | [113] | 517 |
| USA | | Texas | MT | - | [114] | 518 |
| USA | | Texas | Y | - | [114] | 519 |
| USA | | Vermont | Y | - | [113] | 520 |
| USA | | Virgiana | MT | - | [114] | 521 |
| USA | | Virgiana | Y | - | [114] | 522 |
| USA | | Virginia | Y | - | [113] | 523 |
| USA | | Washington | MT | - | [114] | 524 |
| Venezuela | | Caracas Private clinic | MT | - | [118] | 525 |
| Venezuela | | Caracas Private clinic | Y | - | [118] | 526 |
| Venezuela | | Caracas Public clinic | MT | - | [118] | 527 |
| Venezuela | | Caracas Public clinic | Y | - | [118] | 528 |
| Venezuela | | Curiepe | MT | - | [80] | 529 |
| Venezuela | | Curiepe | Y | - | [80] | 530 |
| Venezuela | | Panaquire | MT | - | [80] | 531 |
| Venezuela | | Panaquire | Y | - | [80] | 532 |

**References**

1. Corte-Real HB, Macaulay VA, Richards MB, Hariti G, Issad MS, Cambon-Thomsen A, Papiha S, Bertranpetit J, Sykes BC: **Genetic diversity in the Iberian Peninsula determined from mitochondrial sequence analysis**. *Ann Hum Genet* 1996, **60**(Pt 4):331-350.

2. Robino C, Crobu F, Di Gaetano C, Bekada A, Benhamamouch S, Cerutti N, Piazza A, Inturri S, Torre C: **Analysis of Y-chromosomal SNP haplogroups and STR haplotypes in an Algerian population sample**. *Int J Legal Med* 2008, **122**(3):251-255.

3. Beleza S, Gusmao L, Amorim A, Carracedo A, Salas A: **The genetic legacy of western Bantu migrations**. *Hum Genet* 2005, **117**(4):366-375.

4. Coelho M, Sequeira F, Luiselli D, Beleza S, Rocha J: **On the edge of Bantu expansions: mtDNA, Y chromosome and lactase persistence genetic variation in southwestern Angola**. *BMC Evol Biol* 2009, **9**:80.

5. Plaza S, Salas A, Calafell F, Corte-Real F, Bertranpetit J, Carracedo A, Comas D: **Insights into the western Bantu dispersal: mtDNA lineage analysis in Angola**. *Hum Genet* 2004, **115**(5):439-447.

6. Rowold DJ, Luis JR, Terreros MC, Herrera RJ: **Mitochondrial DNA geneflow indicates preferred usage of the Levant Corridor over the Horn of Africa passageway**. *J Hum Genet* 2007, **52**(5):436-447.

7. Luis JR, Rowold DJ, Regueiro M, Caeiro B, Cinnioglu C, Roseman C, Underhill PA, Cavalli-Sforza LL, Herrera RJ: **The Levant versus the Horn of Africa: evidence for bidirectional corridors of human migrations**. *Am J Hum Genet* 2004, **74**(3):532-544.

8. Vigilant L, Stoneking M, Harpending H, Hawkes K, Wilson AC: **African populations and the evolution of human mitochondrial DNA**. *Science* 1991, **253**(5027):1503-1507.

9. Lind JM, Hutcheson-Dilks HB, Williams SM, Moore JH, Essex M, Ruiz-Pesini E, Wallace DC, Tishkoff SA, O'Brien SJ, Smith MW: **Elevated male European and female African contributions to the genomes of African American individuals**. *Hum Genet* 2007, **120**(5):713-722.

10. Watson E, Bauer K, Aman R, Weiss G, von Haeseler A, Paabo S: **mtDNA sequence diversity in Africa**. *Am J Hum Genet* 1996, **59**(2):437-444.

11. Cruciani F, Santolamazza P, Shen P, Macaulay V, Moral P, Olckers A, Modiano D, Holmes S, Destro-Bisol G, Coia V *et al*: **A back migration from Asia to sub-Saharan Africa is supported by high-resolution analysis of human Y-chromosome haplotypes**. *Am J Hum Genet* 2002, **70**(5):1197-1214.

12. Goncalves R, Rosa A, Freitas A, Fernandes A, Kivisild T, Villems R, Brehm A: **Y-chromosome lineages in Cabo Verde Islands witness the diverse geographic origin of its first male settlers**. *Hum Genet* 2003, **113**(6):467-472.

13. Brehm A, Pereira L, Bandelt HJ, Prata MJ, Amorim A: **Mitochondrial portrait of the Cabo Verde archipelago: the Senegambian outpost of Atlantic slave trade**. *Ann Hum Genet* 2002, **66**(Pt 1):49-60.

14. Quintana-Murci L, Quach H, Harmant C, Luca F, Massonnet B, Patin E, Sica L, Mouguiama-Daouda P, Comas D, Tzur S *et al*: **Maternal traces of deep common ancestry and asymmetric gene flow between Pygmy hunter-gatherers and Bantu-speaking farmers**. *Proc Natl Acad Sci U S A* 2008, **105**(5):1596-1601.

15. Berniell-Lee G, Calafell F, Bosch E, Heyer E, Sica L, Mouguiama-Daouda P, van der Veen L, Hombert JM, Quintana-Murci L, Comas D: **Genetic and demographic implications of the Bantu expansion: insights from human paternal lineages**. *Mol Biol Evol* 2009, **26**(7):1581-1589.

16. Coia V, Destro-Bisol G, Verginelli F, Battaggia C, Boschi I, Cruciani F, Spedini G, Comas D, Calafell F: **Brief communication: mtDNA variation in North Cameroon: lack of Asian lineages and implications for back migration from Asia to sub-Saharan Africa**. *Am J Phys Anthropol* 2005, **128**(3):678-681.

17. Wood ET, Stover DA, Ehret C, Destro-Bisol G, Spedini G, McLeod H, Louie L, Bamshad M, Strassmann BI, Soodyall H *et al*: **Contrasting patterns of Y chromosome and mtDNA variation in Africa: evidence for sex-biased demographic processes**. *Eur J Hum Genet* 2005, **13**(7):867-876.

18. Ingman M, Kaessmann H, Paabo S, Gyllensten U: **Mitochondrial genome variation and the origin of modern humans**. *Nature* 2000, **408**(6813):708-713.

19. Destro-Bisol G, Coia V, Boschi I, Verginelli F, Caglia A, Pascali V, Spedini G, Calafell F: **The analysis of variation of mtDNA hypervariable region 1 suggests that Eastern and Western Pygmies diverged before the Bantu expansion**. *Am Nat* 2004, **163**(2):212-226.

20. Batini C, Coia V, Battaggia C, Rocha J, Pilkington MM, Spedini G, Comas D, Destro-Bisol G, Calafell F: **Phylogeography of the human mitochondrial L1c haplogroup: genetic signatures of the prehistory of Central Africa**. *Mol Phylogenet Evol* 2007, **43**(2):635-644.

21. Maca-Meyer N, Arnay M, Rando JC, Flores C, Gonzalez AM, Cabrera VM, Larruga JM: **Ancient mtDNA analysis and the origin of the Guanches**. *Eur J Hum Genet* 2004, **12**(2):155-162.

22. Lecerf M, Filali M, Gresenguet G, Ndjoyi-Mbiguino A, Le Goff J, de Mazancourt P, Belec L: **Allele frequencies and haplotypes of eight Y-short tandem repeats in Bantu population living in Central Africa**. *Forensic Sci Int* 2007, **171**(2-3):212-215.

23. Soodyall H, Vigilant L, Hill AV, Stoneking M, Jenkins T: **mtDNA control-region sequence variation suggests multiple independent origins of an "Asian-specific" 9-bp deletion in sub-Saharan Africans**. *Am J Hum Genet* 1996, **58**(3):595-608.

24. Cerny V, Salas A, Hajek M, Zaloudkova M, Brdicka R: **A bidirectional corridor in the Sahel-Sudan belt and the distinctive features of the Chad Basin populations: a history revealed by the mitochondrial DNA genome**. *Ann Hum Genet* 2007, **71**(Pt 4):433-452.

25. Krings M, Salem AE, Bauer K, Geisert H, Malek AK, Chaix L, Simon C, Welsby D, Di Rienzo A, Utermann G *et al*: **mtDNA analysis of Nile River Valley populations: A genetic corridor or a barrier to migration?** *Am J Hum Genet* 1999, **64**(4):1166-1176.

26. Maca-Meyer N, Gonzalez AM, Larruga JM, Flores C, Cabrera VM: **Major genomic mitochondrial lineages delineate early human expansions**. *BMC Genet* 2001, **2**:13.

27. Coudray C, Olivieri A, Achilli A, Pala M, Melhaoui M, Cherkaoui M, El-Chennawi F, Kossmann M, Torroni A, Dugoujon JM: **The complex and diversified mitochondrial gene pool of Berber populations**. *Ann Hum Genet* 2009, **73**(2):196-214.

28. Stevanovitch A, Gilles A, Bouzaid E, Kefi R, Paris F, Gayraud RP, Spadoni JL, El-Chenawi F, Beraud-Colomb E: **Mitochondrial DNA sequence diversity in a sedentary population from Egypt**. *Ann Hum Genet* 2004, **68**(Pt 1):23-39.

29. Mateu E, Comas D, Calafell F, Perez-Lezaun A, Abade A, Bertranpetit J: **A tale of two islands: population history and mitochondrial DNA sequence variation of Bioko and Sao Tome, Gulf of Guinea**. *Ann Hum Genet* 1997, **61**(Pt 6):507-518.

30. Pinto F, Gonzalez AM, Hernandez M, Larruga JM, Cabrera VM: **Genetic relationship between the Canary Islanders and their African and Spanish ancestors inferred from mitochondrial DNA sequences**. *Ann Hum Genet* 1996, **60**(Pt 4):321-330.

31. Arroyo-Pardo E, Gusmao L, Lopez-Parra AM, Baeza C, Mesa MS, Amorim A: **Genetic variability of 16 Y-chromosome STRs in a sample from Equatorial Guinea (Central Africa)**. *Forensic Sci Int* 2005, **149**(1):109-113.

32. Semino O, Santachiara-Benerecetti AS, Falaschi F, Cavalli-Sforza LL, Underhill PA: **Ethiopians and Khoisan share the deepest clades of the human Y-chromosome phylogeny**. *Am J Hum Genet* 2002, **70**(1):265-268.

33. Kivisild T, Reidla M, Metspalu E, Rosa A, Brehm A, Pennarun E, Parik J, Geberhiwot T, Usanga E, Villems R: **Ethiopian mitochondrial DNA heritage: tracking gene flow across and around the gate of tears**. *Am J Hum Genet* 2004, **75**(5):752-770.

34. Rosa A, Ornelas C, Jobling MA, Brehm A, Villems R: **Y-chromosomal diversity in the population of Guinea-Bissau: a multiethnic perspective**. *BMC Evol Biol* 2007, **7**:124.

35. Brandstatter A, Peterson CT, Irwin JA, Mpoke S, Koech DK, Parson W, Parsons TJ: **Mitochondrial DNA control region sequences from Nairobi (Kenya): inferring phylogenetic parameters for the establishment of a forensic database**. *Int J Legal Med* 2004, **118**(5):294-306.

36. Immel A-D, Erhuma M, Mustafa T, Kleiber M, Klintshar M: **Y-chromosomal STR haplotypes in an Arab population from Lybia**. *International Congress Series* 2006, **1288**:156-158.

37. Ottoni C, Martinez-Labarga C, Loogvali EL, Pennarun E, Achilli A, De Angelis F, Trucchi E, Contini I, Biondi G, Rickards O: **First genetic insight into Libyan Tuaregs: a maternal perspective**. *Ann Hum Genet* 2009, **73**(Pt 4):438-448.

38. Tofanelli S, Bertoncini S, Castri L, Luiselli D, Calafell F, Donati G, Paoli G: **On the origins and admixture of Malagasy: new evidence from high-resolution analyses of paternal and maternal lineages**. *Mol Biol Evol* 2009, **26**(9):2109-2124.

39. Hurles ME, Sykes BC, Jobling MA, Forster P: **The dual origin of the Malagasy in Island Southeast Asia and East Africa: evidence from maternal and paternal lineages**. *Am J Hum Genet* 2005, **76**(5):894-901.

40. Ely B, Wilson JL, Jackson F, Jackson BA: **African-American mitochondrial DNAs often match mtDNAs found in multiple African ethnic groups**. *BMC Biol* 2006, **4**:34.

41. Goncalves R, Freitas A, Branco M, Rosa A, Fernandes AT, Zhivotovsky LA, Underhill PA, Kivisild T, Brehm A: **Y-chromosome lineages from Portugal, Madeira and Acores record elements of Sephardim and Berber ancestry**. *Ann Hum Genet* 2005, **69**(Pt 4):443-454.

42. Rando JC, Pinto F, Gonzalez AM, Hernandez M, Larruga JM, Cabrera VM, Bandelt HJ: **Mitochondrial DNA analysis of northwest African populations reveals genetic exchanges with European, near-eastern, and sub-Saharan populations**. *Ann Hum Genet* 1998, **62**(Pt 6):531-550.

43. Brakez Z, Bosch E, Izaabel H, Akhayat O, Comas D, Bertranpetit J, Calafell F: **Human mitochondrial DNA sequence variation in the Moroccan population of the Souss area**. *Ann Hum Biol* 2001, **28**(3):295-307.

44. Salas A, Richards M, De la Fe T, Lareu MV, Sobrino B, Sanchez-Diz P, Macaulay V, Carracedo A: **The making of the African mtDNA landscape**. *Am J Hum Genet* 2002, **71**(5):1082-1111.

45. Pereira L, Gusmao L, Alves C, Amorim A, Prata MJ: **Bantu and European Y-lineages in Sub-Saharan Africa**. *Ann Hum Genet* 2002, **66**(Pt 5-6):369-378.

46. Fujihara J, Yuasa I, Muro T, Iida R, Tsubota E, Nakamura H, Imamura S, Yasuda T, Takeshita H: **Allele frequencies and haplotypes for 28 Y-STRs in Ovambo population**. *Leg Med (Tokyo)* 2009, **11**(4):205-208.

47. Pritchard JK, Seielstad MT, Perez-Lezaun A, Feldman MW: **Population growth of human Y chromosomes: a study of Y chromosome microsatellites**. *Mol Biol Evol* 1999, **16**(12):1791-1798.

48. Lum JK, Rickards O, Ching C, Cann RL: **Polynesian mitochondrial DNAs reveal three deep maternal lineage clusters**. *Hum Biol* 1994, **66**(4):567-590.

49. He M, Gitschier J, Zerjal T, de Knijff P, Tyler-Smith C, Xue Y: **Geographical affinities of the HapMap samples**. *PLoS One* 2009, **4**(3):e4684.

50. Dubut V, Murail P, Pech N, Thionville MD, Cartault F: **Inter- and extra-Indian admixture and genetic diversity in reunion island revealed by analysis of mitochondrial DNA**. *Ann Hum Genet* 2009, **73**(Pt 3):314-334.

51. Berniell-Lee G, Plaza S, Bosch E, Calafell F, Jourdan E, Cesari M, Lefranc G, Comas D: **Admixture and sexual bias in the population settlement of La Reunion Island (Indian Ocean)**. *Am J Phys Anthropol* 2008, **136**(1):100-107.

52. Trovoada MJ, Tavares L, Gusmao L, Alves C, Abade A, Amorim A, Prata MJ: **Dissecting the genetic history of Sao Tome e Principe: a new window from Y-chromosome biallelic markers**. *Ann Hum Genet* 2007, **71**(Pt 1):77-85.

53. Trovoada MJ, Pereira L, Gusmao L, Abade A, Amorim A, Prata MJ: **Pattern of mtDNA variation in three populations from Sao Tome e Principe**. *Ann Hum Genet* 2004, **68**(Pt 1):40-54.

54. Graven L, Passarino G, Semino O, Boursot P, Santachiara-Benerecetti S, Langaney A, Excoffier L: **Evolutionary correlation between control region sequence and restriction polymorphisms in the mitochondrial genome of a large Senegalese Mandenka sample**. *Mol Biol Evol* 1995, **12**(2):334-345.

55. Jackson BA, Wilson JL, Kirbah S, Sidney SS, Rosenberger J, Bassie L, Alie JA, McLean DC, Garvey WT, Ely B: **Mitochondrial DNA genetic diversity among four ethnic groups in Sierra Leone**. *Am J Phys Anthropol* 2005, **128**(1):156-163.

56. Monson KL, Miller KWP, Wilson MR, Dizinno JA, Budowle B: **The mtDNA population database: an integrated software and database resource for forensic comparison**. *Forensic Sci Comm* 2002, **4**.

57. Hallenberg C, Simonsen B, Sanchez J, Morling N: **Y-chromosome STR haplotypes in Somalis**. *Forensic Sci Int* 2005, **151**(2-3):317-321.

58. Cerny V, Pereira L, Kujanova M, Vasikova A, Hajek M, Morris M, Mulligan CJ: **Out of Arabia-the settlement of island Soqotra as revealed by mitochondrial and Y chromosome genetic diversity**. *Am J Phys Anthropol* 2009, **138**(4):439-447.

59. Tishkoff SA, Gonder MK, Henn BM, Mortensen H, Knight A, Gignoux C, Fernandopulle N, Lema G, Nyambo TB, Ramakrishnan U *et al*: **History of click-speaking populations of Africa inferred from mtDNA and Y chromosome genetic variation**. *Mol Biol Evol* 2007, **24**(10):2180-2195.

60. Hey J: **Mitochondrial and nuclear genes present conflicting portraits of human origins**. *Mol Biol Evol* 1997, **14**(2):166-172.

61. Chen YS, Olckers A, Schurr TG, Kogelnik AM, Huoponen K, Wallace DC: **mtDNA variation in the South African Kung and Khwe-and their genetic relationships to other African populations**. *Am J Hum Genet* 2000, **66**(4):1362-1383.

62. Mishmar D, Ruiz-Pesini E, Golik P, Macaulay V, Clark AG, Hosseini S, Brandon M, Easley K, Chen E, Brown MD *et al*: **Natural selection shaped regional mtDNA variation in humans**. *Proc Natl Acad Sci U S A* 2003, **100**(1):171-176.

63. Hassan HY, Underhill PA, Cavalli-Sforza LL, Ibrahim ME: **Y-chromosome variation among Sudanese: restricted gene flow, concordance with language, geography, and history**. *Am J Phys Anthropol* 2008, **137**(3):316-323.

64. Knight A, Underhill PA, Mortensen HM, Zhivotovsky LA, Lin AA, Henn BM, Louis D, Ruhlen M, Mountain JL: **African Y chromosome and mtDNA divergence provides insight into the history of click languages**. *Curr Biol* 2003, **13**(6):464-473.

65. Turchi C, Buscemi L, Giacchino E, Onofri V, Fendt L, Parson W, Tagliabracci A: **Polymorphisms of mtDNA control region in Tunisian and Moroccan populations: an enrichment of forensic mtDNA databases with Northern Africa data**. *Forensic Sci Int Genet* 2009, **3**(3):166-172.

66. Frigi S, Pereira F, Pereira L, Yacoubi B, Gusmao L, Alves C, Khodjet el Khil H, Cherni L, Amorim A, El Gaaied A: **Data for Y-chromosome haplotypes defined by 17 STRs (AmpFLSTR Yfiler) in two Tunisian Berber communities**. *Forensic Sci Int* 2006, **160**(1):80-83.

67. Ayadi I, Ammar-Keskes L, Rebai A: **Haplotypes for 13 Y-chromosomal STR loci in South Tunisian population (Sfax region)**. *Forensic Sci Int* 2006, **164**(2-3):249-253.

68. Horai S, Hayasaka K: **Intraspecific nucleotide sequence differences in the major noncoding region of human mitochondrial DNA**. *Am J Hum Genet* 1990, **46**(4):828-842.

69. Castri L, Tofanelli S, Garagnani P, Bini C, Fosella X, Pelotti S, Paoli G, Pettener D, Luiselli D: **mtDNA variability in two Bantu-speaking populations (Shona and Hutu) from Eastern Africa: implications for peopling and migration patterns in sub-Saharan Africa**. *Am J Phys Anthropol* 2009, **140**(2):302-311.

70. Bobillo MC, Zimmermann B, Sala A, Huber G, Rock A, Bandelt HJ, Corach D, Parson W: **Amerindian mitochondrial DNA haplogroups predominate in the population of Argentina: towards a first nationwide forensic mitochondrial DNA sequence database**. *Int J Legal Med* 2009.

71. Salas A, Jaime JC, Alvarez-Iglesias V, Carracedo A: **Gender bias in the multiethnic genetic composition of central Argentina**. *J Hum Genet* 2008, **53**(7):662-674.

72. Dipierri JE, Alfaro E, Martinez-Marignac VL, Bailliet G, Bravi CM, Cejas S, Bianchi NO: **Paternal directional mating in two Amerindian subpopulations located at different altitudes in northwestern Argentina**. *Hum Biol* 1998, **70**(6):1001-1010.

73. Martinez Marignac VL, Bertoni B, Parra EJ, Bianchi NO: **Characterization of admixture in an urban sample from Buenos Aires, Argentina, using uniparentally and biparentally inherited genetic markers**. *Hum Biol* 2004, **76**(4):543-557.

74. Monsalve MV, Hagelberg E: **Mitochondrial DNA polymorphisms in Carib people of Belize**. *Proc Biol Sci* 1997, **264**(1385):1217-1224.

75. Ribeiro GG, De Lima RR, Wiezel CE, Ferreira LB, Sousa SM, Rocha DM, Canas Mdo C, Nardelli-Costa J, Klautau-Guimaraes Mde N, Simoes AL *et al*: **Afro-derived Brazilian populations: male genetic constitution estimated by Y-chromosomes STRs and AluYAP element polymorphisms**. *Am J Hum Biol* 2009, **21**(3):354-356.

76. Barbosa AB, da Silva LA, Azevedo DA, Balbino VQ, Mauricio-da-Silva L: **Mitochondrial DNA control region polymorphism in the population of Alagoas state, north-eastern Brazil**. *J Forensic Sci* 2008, **53**(1):142-146.

77. Abe-Sandes K, Silva WA, Jr., Zago MA: **Heterogeneity of the Y chromosome in Afro-Brazilian populations**. *Hum Biol* 2004, **76**(1):77-86.

78. Batista dos Santos SE, Rodrigues JD, Ribeiro-dos-Santos AK, Zago MA: **Differential contribution of indigenous men and women to the formation of an urban population in the Amazon region as revealed by mtDNA and Y-DNA**. *Am J Phys Anthropol* 1999, **109**(2):175-180.

79. Silva WA, Bortolini MC, Schneider MP, Marrero A, Elion J, Krishnamoorthy R, Zago MA: **MtDNA haplogroup analysis of black Brazilian and sub-Saharan populations: implications for the Atlantic slave trade**. *Hum Biol* 2006, **78**(1):29-41.

80. Bortolini MC, Da Silva WAJW, De Guerra DC, Remonatto G, Mirandola R, Hutz MH, Weimer TA, Silva MC, Zago MA, Salzano FM: **African-derived South American populations: A history of symmetrical and asymmetrical matings according to sex revealed by bi- and uni-parental genetic markers**. *Am J Hum Biol* 1999, **11**(4):551-563.

81. Ribeiro-dos-Santos AK, Pereira JM, Lobato MR, Carvalho BM, Guerreiro JF, Batista Dos Santos SE: **Dissimilarities in the process of formation of Curiau, a semi-isolated Afro-Brazilian population of the Amazon region**. *Am J Hum Biol* 2002, **14**(4):440-447.

82. Carvalho BM, Bortolini MC, Batista dos Santos SE, Ribeiro-dos-Santos AK: **Mitochondrial DNA mapping of social-biological interactions in Brazilian Amazonian African-descendant populations**. *Genetics and Molecular Biology* 2008, **31**(1):12-22.

83. Alves-Silva J, da Silva Santos M, Guimaraes PE, Ferreira AC, Bandelt HJ, Pena SD, Prado VF: **The ancestry of Brazilian mtDNA lineages**. *Am J Hum Genet* 2000, **67**(2):444-461.

84. Goncalves VF, Carvalho CM, Bortolini MC, Bydlowski SP, Pena SD: **The phylogeography of African Brazilians**. *Hum Hered* 2008, **65**(1):23-32.

85. Ribeiro-dos-Santos AK, Carvalho BM, Feio-dos-Santos AC, dos Santos SE: **Nucleotide variability of HV-I in Afro-descendents populations of the Brazilian Amazon Region**. *Forensic Sci Int* 2007, **167**(1):77-80.

86. Hunemeier T, Carvalho C, Marrero AR, Salzano FM, Junho Pena SD, Bortolini MC: **Niger-Congo speaking populations and the formation of the Brazilian gene pool: mtDNA and Y-chromosome data**. *Am J Phys Anthropol* 2007, **133**(2):854-867.

87. Feio-Dos-Santos AC, Carvalho BM, Batista dos Santos SE, Ribeiro-dos-Santos AK: **Nucleotide variability of HV-I in admixed population of the Brazilian Amazon Region**. *Forensic Sci Int* 2006, **164**(2-3):276-277.

88. Pena SD, Bastos-Rodrigues L, Pimenta JR, Bydlowski SP: **DNA tests probe the genomic ancestry of Brazilians**. *Braz J Med Biol Res* 2009, **42**(10):870-876.

89. Goncalves VF, Prosdocimi F, Santos LS, Ortega JM, Pena SDJ: **Sex-biased gene flow in African Americans but not in Americans Caucasians**. *Genetics and Molecular Research* 2007, **6**(2):256-261.

90. Rocco PP, Morales CG, Moraga MV, Miquel JFP, Nervi FO, Llop ER, Carvallo PS, E. RF: **Genetic composition of the Chilean population: Analysis of mitochondrial DNA polymorphisms**. *Rev méd Chile* 2002, **130**(2):125-131.

91. Cifuentes L, Morales R, Sepulveda D, Jorquera H, Acuna M: **DYS19 and DYS199 loci in a Chilean population of mixed ancestry**. *Am J Phys Anthropol* 2004, **125**(1):85-89.

92. Salas A, Acosta A, Alvarez-Iglesias V, Cerezo M, Phillips C, Lareu MV, Carracedo A: **The mtDNA ancestry of admixed Colombian populations**. *Am J Hum Biol* 2008, **20**(5):584-591.

93. Vergara C, Caraballo L, Mercado D, Jimenez S, Rojas W, Rafaels N, Hand T, Campbell M, Tsai YJ, Gao L *et al*: **African ancestry is associated with risk of asthma and high total serum IgE in a population from the Caribbean Coast of Colombia**. *Hum Genet* 2009, **125**(5-6):565-579.

94. Acosta MA, Blanco-Verea A, Lareu MV, Brion M, Carracedo A: **The genetic male component of two South-Western Colombian populations**. *Forensic Sci Int Genet* 2009, **3**(2):e59-61.

95. Carvajal-Carmona LG, Soto ID, Pineda N, Ortiz-Barrientos D, Duque C, Ospina-Duque J, McCarthy M, Montoya P, Alvarez VM, Bedoya G *et al*: **Strong Amerind/white sex bias and a possible Sephardic contribution among the founders of a population in northwest Colombia**. *Am J Hum Genet* 2000, **67**(5):1287-1295.

96. Rodas C, Gelvez N, Keyeux G: **Mitochondrial DNA studies show asymmetrical Amerindian admixture in Afro-Colombian and Mestizo populations**. *Hum Biol* 2003, **75**(1):13-30.

97. Bedoya G, Montoya P, Garcia J, Soto I, Bourgeois S, Carvajal L, Labuda D, Alvarez V, Ospina J, Hedrick PW *et al*: **Admixture dynamics in Hispanics: a shift in the nuclear genetic ancestry of a South American population isolate**. *Proc Natl Acad Sci U S A* 2006, **103**(19):7234-7239.

98. Mendizabal I, Sandoval K, Berniell-Lee G, Calafell F, Salas A, Martinez-Fuentes A, Comas D: **Genetic origin, admixture, and asymmetry in maternal and paternal human lineages in Cuba**. *BMC Evol Biol* 2008, **8**:213.

99. Tajima A, Hamaguchi K, Terao H, Oribe A, Perrotta VM, Baez CA, Arias JR, Yoshimatsu H, Sakata T, Horai S: **Genetic background of people in the Dominican Republic with or without obese type 2 diabetes revealed by mitochondrial DNA polymorphism**. *J Hum Genet* 2004, **49**(9):495-499.

100. Benn-Torres J, Bonilla C, Robbins CM, Waterman L, Moses TY, Hernandez W, Santos ER, Bennett F, Aiken W, Tullock T *et al*: **Admixture and population stratification in African Caribbean populations**. *Ann Hum Genet* 2008, **72**(Pt 1):90-98.

101. Gonzalez-Andrade F, Sanchez D, Gonzalez-Solorzano J, Gascon S, Martinez-Jarreta B: **Sex-specific genetic admixture of Mestizos, Amerindian Kichwas, and Afro-Ecuadorans from Ecuador**. *Hum Biol* 2007, **79**(1):51-77.

102. Lovo-Gomez J, Blanco-Verea A, Lareu MV, Brion M, Carracedo A: **The genetic male legacy from El Salvador**. *Forensic Sci Int* 2007, **171**(2-3):198-203.

103. Salas A, Lovo-Gomez J, Alvarez-Iglesias V, Cerezo M, Lareu MV, Macaulay V, Richards MB, Carracedo A: **Mitochondrial echoes of first settlement and genetic continuity in El Salvador**. *PLoS One* 2009, **4**(9):e6882.

104. Salas A, Richards M, Lareu MV, Sobrino B, Silva S, Matamoros M, Macaulay V, Carracedo A: **Shipwrecks and founder effects: divergent demographic histories reflected in Caribbean mtDNA**. *Am J Phys Anthropol* 2005, **128**(4):855-860.

105. Matamoros M, Yurrebaso I, Gusmao L, Garcia O: **Population data for 12 Y-chromosome STR loci in a sample from Honduras**. *Leg Med (Tokyo)* 2009, **11**(5):251-255.

106. Rangel-Villalobos H, Munoz-Valle JF, Gonzalez-Martin A, Gorostiza A, Magana MT, Paez-Riberos LA: **Genetic admixture, relatedness, and structure patterns among Mexican populations revealed by the Y-chromosome**. *Am J Phys Anthropol* 2008, **135**(4):448-461.

107. Bonilla C, Bertoni B, Gonzalez S, Cardoso H, Brum-Zorrilla N, Sans M: **Substantial Native American female contribution to the population of Tacuarembo, Uruguay, reveals past episodes of sex-biased gene flow**. *Am J Hum Biol* 2004, **16**(3):289-297.

108. Green LD, Derr JN, Knight A: **mtDNA affinities of the peoples of North-Central Mexico**. *Am J Hum Genet* 2000, **66**(3):989-998.

109. Rodriguez-Delfin LA, Rubin-de-Celis VE, Zago MA: **Genetic diversity in an Andean population from Peru and regional migration patterns of Amerindians in South America: data from Y chromosome and mitochondrial DNA**. *Hum Hered* 2001, **51**(1-2):97-106.

110. Martinez-Cruzado JC, Toro-Labrador G, Viera-Vera J, Rivera-Vega MY, Startek J, Latorre-Esteves M, Roman-Colon A, Rivera-Torres R, Navarro-Millan IY, Gomez-Sanchez E *et al*: **Reconstructing the population history of Puerto Rico by means of mtDNA phylogeographic analysis**. *Am J Phys Anthropol* 2005, **128**(1):131-155.

111. Sans M, Merriwether DA, Hidalgo PC, Bentancor N, Weimer TA, Franco MH, Alvarez I, Kemp BM, Salzano FM: **Population structure and admixture in Cerro Largo, Uruguay, based on blood markers and mitochondrial DNA polymorphisms**. *Am J Hum Biol* 2006, **18**(4):513-524.

112. Sans M, Weimer TA, Franco MH, Salzano FM, Bentancor N, Alvarez I, Bianchi NO, Chakraborty R: **Unequal contributions of male and female gene pools from parental populations in the African descendants of the city of Melo, Uruguay**. *Am J Phys Anthropol* 2002, **118**(1):33-44.

113. Hammer MF, Chamberlain VF, Kearney VF, Stover D, Zhang G, Karafet T, Walsh B, Redd AJ: **Population structure of Y chromosome SNP haplogroups in the United States and forensic implications for constructing Y chromosome STR databases**. *Forensic Sci Int* 2006, **164**(1):45-55.

114. Kayser M, Brauer S, Schadlich H, Prinz M, Batzer MA, Zimmerman PA, Boatin BA, Stoneking M: **Y chromosome STR haplotypes and the genetic structure of U.S. populations of African, European, and Hispanic ancestry**. *Genome Res* 2003, **13**(4):624-634.

115. Parsons TJ: **Mitochondrial DNA Genome Sequencing and SNP Assay Development for Increased Power of Discrimination**. *NCJRS, report of the US Department of Justice* 2006.

116. Parra EJ, Kittles RA, Argyropoulos G, Pfaff CL, Hiester K, Bonilla C, Sylvester N, Parrish-Gause D, Garvey WT, Jin L *et al*: **Ancestral proportions and admixture dynamics in geographically defined African Americans living in South Carolina**. *Am J Phys Anthropol* 2001, **114**(1):18-29.

117. Allard MW, Polanskey D, Miller K, Wilson MR, Monson KL, Budowle B: **Characterization of human control region sequences of the African American SWGDAM forensic mtDNA data set**. *Forensic Sci Int* 2005, **148**(2-3):169-179.

118. Martinez H, Rodriguez-Larralde A, Izaguirre MH, De Guerra DC: **Admixture estimates for Caracas, Venezuela, based on autosomal, Y-chromosome, and mtDNA markers**. *Hum Biol* 2007, **79**(2):201-213.
