# Supplementary figures and images for: The imprint of the Slave Trade in an African American population: mitochondrial DNA, Y chromosome and HTLV-1 analysis in the Noir Marron of French Guiana
Source: BMC Evol Biol. 2010 Oct 19;10:314. doi: 10.1186/1471-2148-10-314 (PMC2973943; doi:10.1186/1471-2148-10-314)

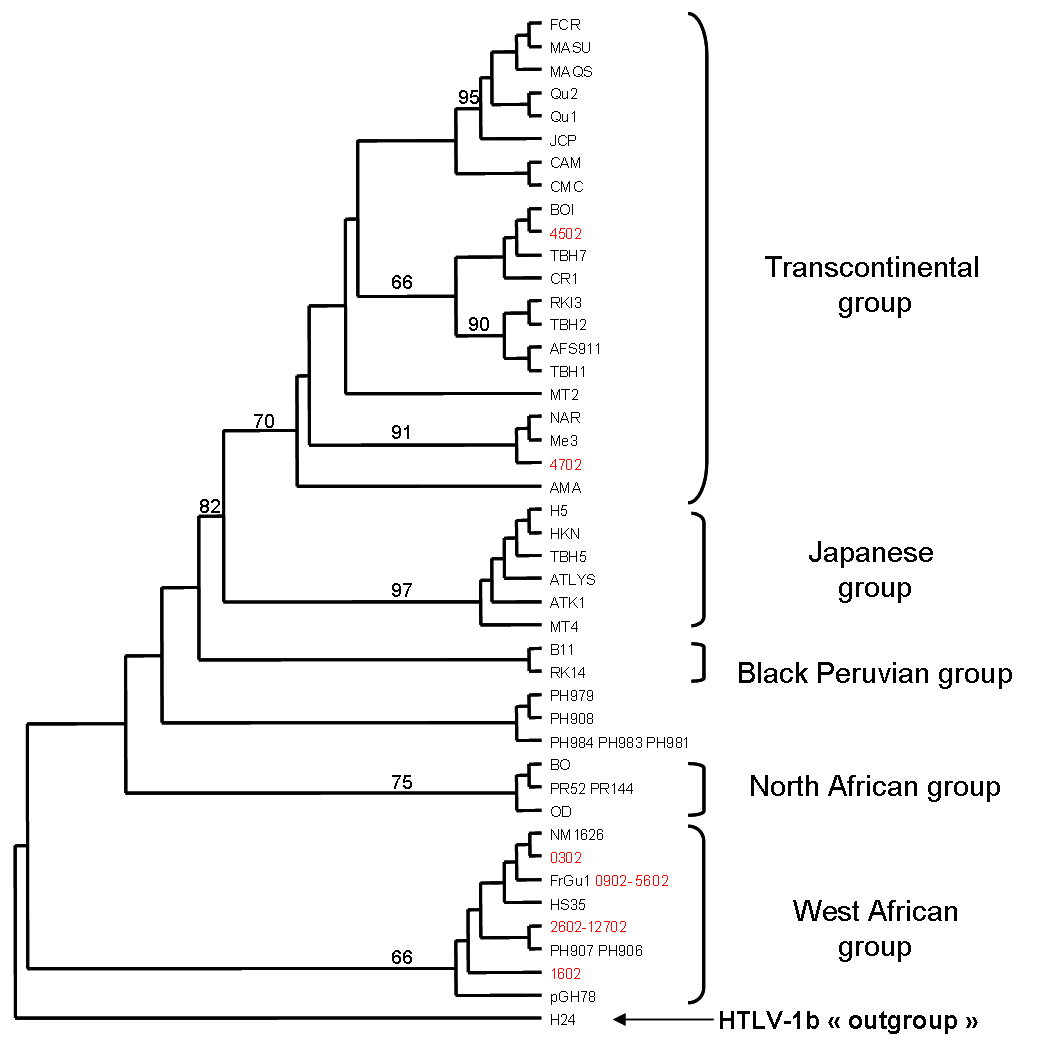

Supplement: Additional file 5 — LTR phylogenetic tree constructed by the neighbour-joining method of HTLV-1 strains in 8 Noir Marron (in red) and HTLV-1 sequences of the database. The Noir Marron data already published are coded "NM". The H24 strain was used as out-group. The HTLV-1 strains were aligned with the DAMBE program (version 4.2.13). The final alignment was submitted to the Modeltest program (version 3.6) to select, according to the Akaike Information Criterion (AIC), the best model to apply to phylogenetic analyses. The selected model was the GTR. Bootstrap support (1,000 replicates) is noted on the branch of the tree. [file 1471-2148-10-314-S5.BMP]
